# Insect phylogeny structures the bacterial communities in the microbiome of psyllids (Hemiptera: Psylloidea) in Aotearoa New Zealand

**DOI:** 10.1371/journal.pone.0285587

**Published:** 2023-05-15

**Authors:** Francesco Martoni, Simon R. Bulman, Alexander M. Piper, Andrew Pitman, Gary S. Taylor, Karen F. Armstrong

**Affiliations:** 1 Bio-Protection Research Centre, Lincoln University, Lincoln, New Zealand; 2 Plant Biosecurity Cooperative Research Centre, University of Canberra, Canberra, ACT, Australia; 3 Agriculture Victoria, AgriBio Centre, Bundoora, VIC, Australia; 4 The New Zealand Institute for Plant & Food Research Ltd, Lincoln, New Zealand; 5 Better Border Biosecurity (B3), Lincoln, New Zealand; 6 Foundation of Arable Research, Hornby, Christchurch, New Zealand; 7 The University of Adelaide, Adelaide, South Australia; 8 Agricultural and Life Sciences Faculty, Lincoln University, Lincoln, New Zealand; University of Helsinki: Helsingin Yliopisto, FINLAND

## Abstract

The bacterial microbiome of psyllids has been studied for decades, with a strong focus on the primary and secondary endosymbionts capable of providing essential amino acids for the insects’ diet and therefore playing a key role in the insects’ ability to radiate on novel plant hosts. Here, we combine metabarcoding analysis of the bacterial communities hosted by psyllids with a multi-gene phylogenetic analysis of the insect hosts to determine what factors influence the bacterial diversity of the psyllids’ microbiomes, especially in the context of the dispersal and evolutionary radiation of these insects in Aotearoa New Zealand. Using multi-gene phylogenetics with COI, 18S and EF-1α sequences from 102 psyllid species, we confirmed for the first time monophyly for all the six genera of native/endemic Aotearoa New Zealand psyllids, with indications that they derive from at least six dispersal events to the country. This also revealed that, after its ancestral arrival, the genus *Powellia* has radiated onto a larger and more diverse range of plants than either *Psylla* or *Ctenarytaina*, which is uncommon amongst monophyletic psyllids globally. DNA metabarcoding of the bacterial 16S gene here represents the largest dataset analysed to date from psyllids, including 246 individuals from 73 species. This provides novel evidence that bacterial diversity across psyllid species is strongly associated with psyllid phylogenetic structure, and to a lesser degree to their host plant association and geographic distribution. Furthermore, while the strongest co-phylogenetic signals were derived from the primary and secondary symbionts, a signal of phylosymbiosis was still retained among the remaining taxa of the bacterial microbiome, suggesting potential vertical transmission of bacterial lineages previously unknown to have symbiotic roles.

## 1. Introduction

Psyllids, also known as jumping plant-lice, are phloem-feeding hemipteran insects belonging to the superfamily Psylloidea (Sternorrhyncha), which is composed of approximately 4000 described species worldwide [1]. The psyllid life cycle includes five immature (nymphal) stages prior to adulthood. Only plant species on which nymphs complete their life cycle are formally considered as “host plants” [2]. As such, psyllids are generally characterised by a strong host plant specificity, with each species usually associated with a single host plant species or a few within the same genus [3–5]. While remaining host specific at a species level, some psyllid genera are comprised of species which amongst them have adapted to a very wide range of hosts. Of note are the 346 species of *Trioza* that are associated with 154 plant genera and 59 plant families [6], and the more than 70 species of *Diaphorina* associated with at least 42 plant genera and 22 plant families worldwide [1]. It is unclear whether there is a greater propensity for host switches within such genera or if the observed host associations are inflated when polyphyly is apparent, as is the case for the *Trioza* [7,8]. Indeed, a recent phylogenetic analysis of the superfamily Psylloidea highlighted substantial genetic variation within various genera, suggesting the need for taxonomical revision of a number of these [9].

Sap-sucking insects rely on their endosymbiotic bacteria for a successful relationship with their host plant, through generation of essential amino acids and vitamins that are deficient in the sap they feed on [10,11]. For this reason, the relationship between many phytophagous hemipteran species and their nutritional symbionts is one of the most extensively studied insect-microbe associations [12–15], amongst which psyllid endosymbionts have seen particular focus [16–20] with an emphasis on their primary symbiont “*Candidatus* Carsonella rudii” (hereafter *Carsonella*). *Carsonella* (Gammaproteobacteria) is a vertically inherited symbiont found in all psyllids and harboured in vesicles within the bacteriome, a specialised abdominal structure [17,20]. This bacterium has a dramatically reduced genome which encodes most, but not all, of the amino acids required by the psyllid host [21,22]. In the psyllids *Ctenarytaina eucalypti* (Aphalaridae) and *Heteropsylla cubana* (Psyllidae) the co-primary Enterobacteriaceae symbionts, which inhabit syncytial cells of the bacteriome, compensate for metabolic pathways absent in *Carsonella* [10,23]. A variety of secondary symbionts with presumptive co-primary functions, including *Arsenophonus*, *Sodalis* and unclassified Enterobacteriaceae species, have been identified in DNA metabarcode data from a diverse collection of psyllids [24–26]. Many secondary symbionts have established long-term stable associations with psyllids, but incongruence between the phylogenies of symbionts and hosts indicate repeated horizontal transmission between psyllid species [18,26]. However, in some species such as *Pachypsylla* spp. and *Heteropsylla texana*, secondary symbionts could not be detected at all, indicating that other mechanisms, such as using horizontally acquired genes for biosynthesis of essential amino acids, or by morphological and physiological alterations at the feeding site [27], may compensate for deficiencies in the *Carsonella* genome [10,23]. In the citrus psyllid *Diaphorina citri*, a central multinucleate syncytium of the bacteriome contains a co-primary symbiont, *Candidatus* Profftella armatura (Betaproteobacteria) [28] which, like *Carsonella*, has a heavily reduced genome indicative of vertical transmission. *Candidatus* Profftella has however been shown to play a role in defence against natural enemies rather than in host nutrition [29].

Understanding the influence of the bacterial microbiome on the insects’ plant host choice has important implications for our fundamental ecological understanding, but also for plant protection if endosymbionts facilitate the ability of an insect to diversify its diet and thereby improve its invasive potential [30]. To appreciate the relevance of bacterial microflora and host-plant associations, an accurate understanding of psyllid phylogenetic relationships is needed. For example, the most recent study of Kwak and colleagues [26] was only made possible by prior phylogenetic works [9,31] that enabled them to describe the insect-microbe associations, including novel ones relevant to economically important plants, and to discover a new potentially pathogenic *Ca*. Liberibacter species.

Studies such as these that compare the insect phylogenetics with their microbial communities have led to the creation of the term ‘phylosymbiosis’, proposed to indicate ‘microbial community relationships that recapitulate the phylogeny of their host’ [32]. Indeed, microbiome composition across several insect groups has been found to correlate with both insect phylogeny and diet [33,34]. However, since closely related psyllids species also feed on closely related plants, establishing a causative link between specific bacteria and the insects’ ability to feed on certain plants can be challenging [35]. The psyllid fauna of Aotearoa New Zealand, home to more than 120 psyllid species—of which only 74 are fully described [36–38]—provides a unique case study to test these insect-bacteria-plant relationships. These species belong to 24 genera and six families, with the majority of endemic species falling within the genera *Powellia* [39], *Ctenarytaina* and *Psylla* [36]. Since their arrival, these genera have speciated on the archipelago but developed different breadths of association with regard to their host plants. *Psylla* and most species of *Ctenarytaina* are associated with single plant families whereas *Powellia* are hosted by more than 50 plant species across 20 genera over 12 divergent families [40,41].

In this study, we set out to identify factors that have influenced the microbiome composition of psyllids and the evolution of their symbiotic relationship by focusing on the psyllid fauna of Aotearoa New Zealand following their arrival and subsequent adaptation to novel host plants there [40,42]. Our primary hypothesis was that microbiome composition in psyllids is primarily linked to their plant-hosts’ phylogenetic relationships, as it has been reported for the primary symbiont [17,20] and some of the secondary symbionts [18,24]. Since primary and secondary symbionts are known to show a strong co-phylogenetic signal with their insect hosts, our secondary hypothesis was that once these bacterial lineages are removed, other factors—such as the host plant relationships or the geographic distribution—may have a more important role in shaping the microbiome composition. This secondary hypothesis aimed to cast some light on the role played by the environment on the insects’ evolutionary radiation throughout the islands of Aotearoa New Zealand. To test the primary hypothesis, a multi-gene sequence dataset was compiled to determine the phylogenetic structure of the psyllids of Aotearoa New Zealand, evaluate monophyly for each of the three largest native genera, and predict the ancestral psyllid lineages. With that established, we then analysed the bacterial microbiome composition of each species using 16S metabarcoding to investigate whether microbiome composition could be explained by psyllid phylogeny, geographic location, and/or host plant associations.

## 2. Materials and methods

### 2.1. Psyllid DNA sequences

The psyllid specimens used for this study are the same that had been collected and PCR amplified for previous works [36,42,43]. There, DNA was extracted from individual whole psyllids using a cetyltrimethylammonium bromide (CTAB) protocol [44] and then used in this study to generate new sequences following the same methods as Martoni et al., [36]. This added a further 14 *cytochrome oxidase* gene subunit I (COI) sequences to the dataset from Martoni et al., [43] and an additional EF-1α sequence for *Atmetocranium myersi* (S1 Table). In addition, 188 544-bp partial sequence of the small ribosomal subunit (18S) were isolated from single specimens using the primers 18S_F (CTGGTTGATCCTGCCAGAGT; [45]) and 18S_Rmod (ACCAGACTTGCCCTCCAAT; modified in this study from another work [45]). PCR for the 18S gene followed the protocol used for COI in a previous work [36].

The dataset was supplemented by 443 partial sequences of the psyllid COI and 20 partial sequences of the psyllid *Elongation Factor* 1-α (EF-1α) retrieved from GenBank (S1 Table). These included more than 430 COI and 20 EF-1α sequences previously generated from individual adult psyllids from Aotearoa New Zealand and Australia [36,43], and sequences for a few exotic species from the United States of America and Europe, as additional representatives of families present in Aotearoa New Zealand.

The final dataset available for phylogenetic analysis included 458 COI sequences, 188 18S sequences and 23 EF-1α sequences, belonging to a total of 102 psyllid species and an aphid species, *Acyrthosiphon pisum*, used as outgroup (S1 Table). This includes 90 of the 120 taxa present in Aotearoa New Zealand from across all six families there. Most species are represented by sequences from several populations.

### 2.2. Psyllid phylogenetic analysis

Alignments of each gene region (COI, 18S and EF-1α) were generated using MEGA version X [46]. The best substitution model for each gene alignment was calculated in MEGA X using the Bayesian information criterion (BIC, [47]). The General Time-Reversible (GTR) model [48] was used for COI, the Tamura and Nei (TN93, [49]) model for 18S and the Hasegawa, Kishino, Yano (HKY) model [50] for EF-1α. A three-gene species tree was developed using the package Starbeast (*BEAST, [51]) in BEAST v2.5.1, with the Markov Chain Monte Carlo (MCMC) method [52–54] and multiple chains of 1 billion replicates each, logging parameters every 10,000 replicates.

The Aotearoa New Zealand native psyllid species delimitation used to infer the species tree had been presented elsewhere, using an integrative taxonomy approach [36]. This included 21 undescribed species labelled with a letter (e.g., *Psylla carmichaeliae* “sp.A”) [36] and another eight unpublished species labelled after a locality (e.g., *Powellia* “Massey”) [40]. For the purpose of this study, and for consistency in the use of the term, also the undescribed taxa are referred to as a “species”.

Each model was selected together with a gamma distribution rate of 4. The mitochondrial gene COI was set to a 0.5 ploidy compared to the 2.0 for both 18S and EF-1α, as suggested for multi-gene analyses [55]. The software Tracer v1.7 [56] was used for visualization and diagnostics of the MCMC output, including the effective sample size (ESS) statistics. This confirmed that the Markov chain had reached convergence and the resulting posterior ESS was >>200 (508), with the tree likelihood ESS for each tree being 1151 (18S), 587 (COI) and 512 (EF-1α). LogCombiner was used to subsample the number of trees from 500,000 to 100,000. TreeAnnotator [52,53] was used to summarize the information in a single tree and to set a 10% burn-in based on the information visualized with Tracer. The resulting species tree was drawn using FigTree v1.4.3 [57].

### 2.3. Bacterial microbiome sequencing and bioinformatics

The V3 -V4 region of the bacterial 16S ribosomal RNA gene was amplified from 246 individual adult psyllids, including technical replicates for 31 of them, for a total of 277 samples (S2 Table). These individual psyllids were the same used for the isolation of the psyllid genes (See section 2.1). PCR amplification was conducted using S-D-Bact-0341-b-S-17 and S-D-Bact-0785-a-A-21 primers [58], modified with Illumina adapters. PCR cycling was performed using an initial denaturation at 95°C for 3 min, followed by 25 cycles of 95°C denaturation for 30 s, 55°C annealing for 30 s and 72°C elongation for 30 s. Amplicons were purified using the Agencourt® AMPure® XP kit (Beckman Coulter, California, United States). Samples were indexed and sequenced on an Illumina MiSeq platform using 2 x 300 bp reads at New Zealand Genomics Limited (NZGL).

Demultiplexed reads (NCBI SRA acc no: PRJNA805017) were trimmed of primers using BBDuK in BBTools v38.01 [59]. Any sequences containing ambiguous ‘N’ bases or with >2 expected error [60] in the forward read, or >3 in the reverse read were removed. Reverse reads were truncated to 200 bp to minimize the number of reads violating the error filter due to a substantial reduction in quality scores towards the end of the read. Remaining sequences were denoised using DADA2 v1.16 [61,62], with the error model determined separately for each sequencing run using the “pseudo-pooling” mode. The Amplicon Sequence Variants (ASVs) inferred separately from each sequencing run were merged into a single table and any chimeric sequences removed de-novo using the removeBimeraDenovo function. Hierarchical taxonomy was assigned to the 3,881 ASVs to the lowest rank possible with a minimum bootstrap support of 60% using the IDTAXA algorithm [63] trained on the SILVA v138 reference database [64,65]. This was followed by extra species level assignment using exact matching between the sequences reference database [66]. ASVs classified as chloroplast or mitochondrial were filtered out, then technical replicates were merged and any samples with <1,000 remaining reads were removed from the datasets (S1A Fig). The remaining 3,763 ASVs were aligned with 8,148 nearest neighbour sequences from the SILVA 138 database using SINA v1.7.0 [67]. A Maximum Likelihood (ML) bacterial phylogenetic tree was inferred from the alignment using FastTree v2.1.11 [68] with the General Time-Reversible (GTR) model [48] and CAT approximation of rate heterogeneity across sites. Local branch support values were calculated using the default method of resampling the site likelihoods 1,000 times and conducting a Shimodaira Hasegawa-like test. The resulting tree was time scaled and made ultrametric via congruification [69] with the dated SILVA 16S 97% similarity reference tree from a previous work [70] using 2,862 shared tips (nearest neighbour sequences obtained from the SILVA database) and the *geiger* v2.0.7 R package [71].

### 2.4. Statistical analysis

#### 2.4.1 Microbiome diversity

The observed richness of ASVs and the Shannon index [72] were calculated using the R package phyloseq v1.36 [73]. Phylogenetic diversity [74] was calculated with the picante v1.8.2 R package [75]. Differences in α-diversity between species were compared using Analysis of Variance (ANOVA), with terms specified in the order of sequence run, psyllid family, genus, and species to marginalise the effects of technical variation before partitioning the remaining variance into that accounted for by phylogeny. To ensure robustness of ASV comparisons the different sequencing depths between samples, ANOVAs were also conducted with the data rarefied to the sequencing depth of the lowest sample (1,352 reads).

To assess compositional differences in microbial communities (β-diversity), samples were centre log-ratio (CLR) transformed to normalise abundance data [76] with zeroes imputed via Bayesian-multiplicative replacement with the zCompositions v1.3.4 R package [77]. Permutational Multivariate Analysis of Variance Using Distance Matrices (PERMANOVA; adonis2 function) [78] and PERMDISP tests of multivariate homogeneity of group dispersions [79] were conducted on the Euclidean distances of CLR-transformed microbial abundances (Aitchison distance) [80] with 999 permutations using the vegan v2.5.7 R package [81]. As with the ANOVA, terms were structured sequentially to account for technical variation before partitioning the variance along the phylogeny. During the specimen collection some psyllid specimens of different species were collected from the same individual host plant at the same timepoint. Therefore, to further evaluate the differential influence of psyllid species and host plant, differences in β-diversity between these specimens alone was compared using PERMANOVA tests and the number of overlapping ASVs calculated.

#### 2.4.2 Phylosymbiosis

Mantel tests of Pearson’s correlation [82] were conducted between the microbial Aitchison distance matrix and distance matrices of psyllid phylogenetic distance, plant phylogenetic distance or geographic distance using the ecodist v2.0.7 R package [83]. Pairwise phylogenetic distances were calculated from the psyllid phylogenetic tree using the cophenetic.phylo function from the ape v5.5 R package [84], and made Euclidean by taking the element wise square root [85]. Psyllid host plant records [36] were used to retrieve an exemplar phylogenetic tree with phylomatic [86] as implemented in the brranching v0.7 R package [87] and pairwise distances generated from branch lengths. To obtain a geographic distance matrix, pairwise Great Circle distances were calculated between latitude and longitude coordinates from collection locations for each specimen using the sp v1.4.5 R package [88]. Mantel and Partial Mantel tests were assessed for significance against 999 permutations of the rows and columns of each dissimilarity matrix and 95% confidence intervals were obtained from 1,000 bootstrap resamples.

#### 2.4.3 Co-phylogeny

Due to the inability to assign some putative secondary symbionts to lower ranks of taxonomy, a taxonomy free approach was used to assess co-phylogeny between the main three psyllid genera (*Psylla*, *Powellia*, *Ctenarytaina*) and their core microbiome. To identify the core microbiome members for these genera, all observations below 0.01% abundance were first removed to control for any influence of index-switching cross contamination, then the whole-microbiome phylogeny was cut 6.8 x 10^8^ years from the tips to create phylogenetically derived OTUs. A range of cut thresholds were explored, with the 6.8 x 10^8^ years threshold selected as it produced biologically meaningful groupings that generally approximated the family level and captured major symbiont lineages such as *Carsonella* in their own monophyletic clusters. To minimise false positives, the core microbiome was considered those OTUs present in at least 25% of one of the three main genera, with more than five unique ASVs. Co-phylogeny was then investigated between these core microbiome members and their hosts using the Procrustean Approach to Co-phylogeny (PACo) v0.4.2 and ParaFit algorithms [89–91], analysing each of the main genera and core OTU lineages separately. These distance-based approaches do not directly compare the topology of the host and microbe trees and instead compare principal coordinate transformed distances between taxa, making them less sensitive to the effects of topological uncertainty and polytomies in either tree, and able to handle symbionts associating with multiple hosts, and vice-versa. The input ‘host’ and ‘symbiont’ distance matrices were derived from the psyllid species tree and best fit ML tree of the bacteria respectively by taking the pairwise patristic distance (sum of the branch lengths of the branches that link two terminal nodes), which were then transformed using the element-wise square root to eliminate the problem of negative eigenvalues when phylogenetic distance matrices are not Euclidean [85]. Global significance of co-phylogenetic congruence was assessed against 100,000 random permutations of the psyllid-bacteria associations using the ‘quasiswap’ method for PACo, a conservative model which does not assume that the microbe phylogeny tracks the host or vice-versa, and the similar default method for ParaFit. Cophylogenetic associations were considered significant if the p-values were below an alpha of 0.05 following a Bonferroni correction for multiple testing. All statistical analyses above were conducted within the R v4.1 statistical programming environment [92] using tidyverse v1.3.1 packages [93].

## 3. Results

### 3.1. Evolutionary relationships of the Aotearoa New Zealand psyllids

**Fig 1:**
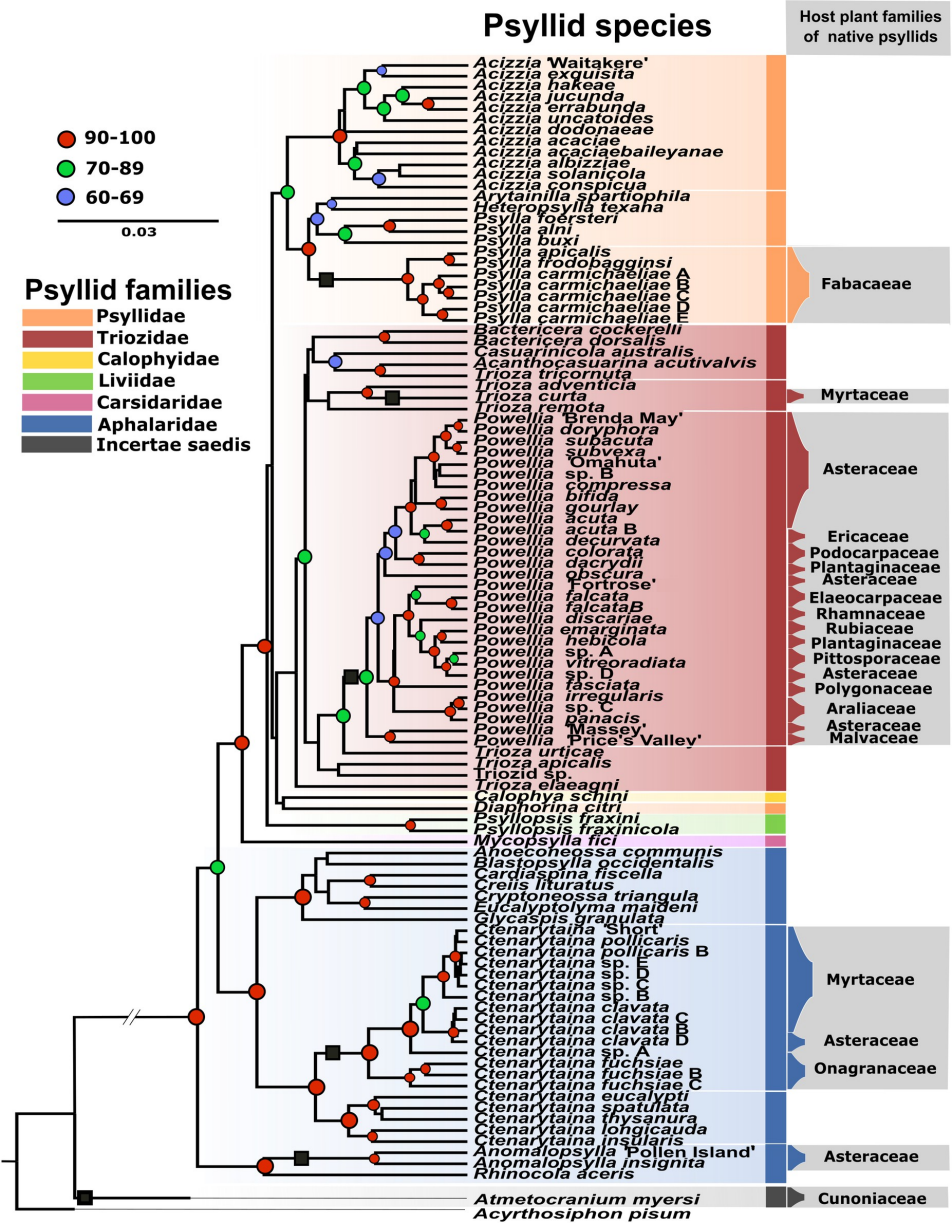
Bayesian Inference species tree of the psylloid taxa included in this study. The species tree was inferred from partial COI, EF-1α and 18S gene sequences and is colour-coded by family (see insert). Aotearoa New Zealand native (mostly endemic) lineages are indicated with black squares on branches, and host plant families are provided in the grey box. Posterior probability values at the nodes are color coded (see insert). Names of undescribed taxa follow previous works [36,40].

In order to test our primary hypothesis that the psyllid phylogenetic relationships shape their microbiome composition, a phylogenetic structure of the Aotearoa New Zealand psyllids was required. A total of 669 DNA sequences across three genes (S1 Table) was used to generate a bayesian inference species tree for 102 psyllid species belonging to 26 genera. The tree (Fig 1) showed higher posterior probability values at levels above species compared with using COI alone [36]. The tree also replicated a number of groupings that have been revealed in other recent phylogenetic studies [9,31], such as a clear spearation of the six families Psyllidae, Triozidae, Calophyidae, Liviidae, Carsidaridae and Aphalaridae. The family Aphalaridae was paraphyletic, in agreement with the results shown elsewhere [31]. Within Aphalaridae (Fig 1, in blue), the separation of the subfamily Rhinocolinae (*Anomalopsylla* + *Rhinocola*) from the subfamily Spondyliaspidinae (all other Aphalarid genera) is well supported, in agreement with what was reported by Cho and colleagues [31].

Of the 103 psyllid species included in this work, 56 are native (mostly endemic) to Aotearoa New Zealand (Fig 1, marked with black squares). These species fell within six generic lineages: seven *Psylla* (Psyllidae), one *Trioza* (Triozidae), 30 *Powellia* (Triozidae), 15 *Ctenarytaina* (Aphalaridae), two *Anomalopsylla* (Aphalaridae), plus one species of *Atmetocranium* (currently ascribed to Calophyidae) (Fig 1, marked with black squares). Systematic relationships of these native taxa relative to the adventive and exotic species are apparent. The Aotearoa New Zealand *Psylla* (Psyllidae) formed a monophyletic group with the nearest relative being *Psylla* species from Europe. Of the Triozidae, the endemic *Trioza curta* was found to have a separate ancestral origin from the other Aotearoa New Zealand triozids, forming a monophyletic association with *T*. *adventicia*, an Australian species hosted by the myrtaceous *Syzygium smithii* [94–96], confirming this species doesn’t belong to the genus *Powellia* [39]. The Australian triozids formed a monophyletic clade, that also included Aotearoa New Zealand’s *T*. *curta*, with very weak affinities amongst species, but in agreement with the hypothesis that this group comprises multiple genera [96]. All 30 species of *Powellia* however clustered into a monophyletic clade supporting it as an Aotearoa New Zealand mostly endemic genus (except for *Powellia vitreoradiata*, which is native). The species grouping closest to *Powellia* was the European *T*. *urticae*, although support for this relationship was low. Indeed, this is mostly due to the limited sampling of species from overseas. When additional species are included in the analysis, *Powellia* groups with *Calinda*, a genus that primarily feeds on Asteraceae [9].

The monophyly of *Powellia* also enabled us to consider the mechanism for speciation that has occurred within Aotearoa New Zealand. Prior to this study, Asteraceae was tentatively thought to be the ancestral plant host for the triozids native to the country, based on the similar morphology of the triozid species associated with this plant group [40,41]. However, the association with Asteraceae was not reported as ancestral within *Powellia* in this study (Fig 1). While one of the earliest diverging species, *Powellia* “Massey” has an Asteraceae host, most of the remaining Asteraceae-inhabiting psyllids were derived from more recent host adoption/speciation events (Fig 1). Furthermore, *Powellia colorata* and *P*. *dacrydii*, which are unusually hosted by a podocarp, *Halocarpus bidwillii*, were confirmed to be most closely related within the broader *Powellia* clade. This confirmed that a very large jump must have occurred from among species hosted by *Hebe* or Asteraceae angiosperms. This suggests that host switches, and not psyllid-plant co-speciations, form the basis of the psyllid radiation in Aotearoa New Zealand. However, considering the close evolutionary relationship between *Powellia* and the Asteraceae-feeding *Calinda* [9], as well as the basal position within *Powellia* of the Asteraceae-feeding *P*. “Massey”, the host jumps discussion above does not preclude the hypothesis that the ancestral *Powellia* was associated with Asteraceae.

The endemic *Ctenarytaina* species (Fig 1, black square; marked as “Endemic” in S1 Table) grouped most closely to five Australian *Ctenarytaina* species (marked as “Introduced” in S1 Table). Within the native *Ctenarytaina* clade, the three taxa associated with *Fuchsia excorticata* (Onagraceae) diverged by 2.1% from all others (hosted by Myrtaceae), with *Ctenarytaina “*sp. A” (hosted by *Olearia*, Asteraceae), branching in between the two groups. At a higher systematic level, the two species of *Anomalopsylla* clustered together with the European species *Rhinocola aceris*; this correctly separated the subfamily Rhinocolinae from the Spondyliaspidinae under which all other aphalarid genera in this dataset are placed. Finally, *Atmetocranium myersi* currently ascribed to the family Calophyidae [8] was clearly separated from *Calophya schini* as the only other species in that family, but also showed no affinity to any other psyllid group in the dataset. This, together with its “highly autapomorphic morphology which makes it difficult to relate to other psylloid groups” [97], suggests that *Atmetocranium* could belong to an entirely new psyllid family.

Of the psyllid species collected in Aotearoa New Zealand but adventive to the country, the most represented clade is the monophyletic genus *Acizzia* (Psyllidae), with 12 species. This includes the species *Acizzia hakeae*, originally described from Aotearoa New Zealand but as yet not recorded from Australia [41], plus the undescribed (presumed endemic to Aotearoa New Zealand) *Acizzia* “Waitakere”. The genus *Ctenarytaina* (Aphalaridae) is monophyletic and clearly separates the five adventive species from the 12 Aotearoa New Zealand endemic ones (Fig 1, black square), with strong support. The single species of Carsidaridae, *Mycopsylla fici*, and the two *Psyllopsis* species of the Liviidae separate their respective families with strong support. All the remaining non-native species, that were not collected in Aotearoa New Zealand, but included in the study to confirm the robustness of the species tree, correctly branched within their assigned families. This is with the exception of *Diaphorina citri* which is separated from the rest of the Psyllidae to which it is currently ascribed [8]. However, it does fall within the larger Psyllidae-Triozidae-Calophyidae-*Diaphorina* (PTCD) clade as previously reported elsewhere [9].

### 3.2. Microbiome composition

#### 3.2.1 Bacterial diversity

**Fig 2:**
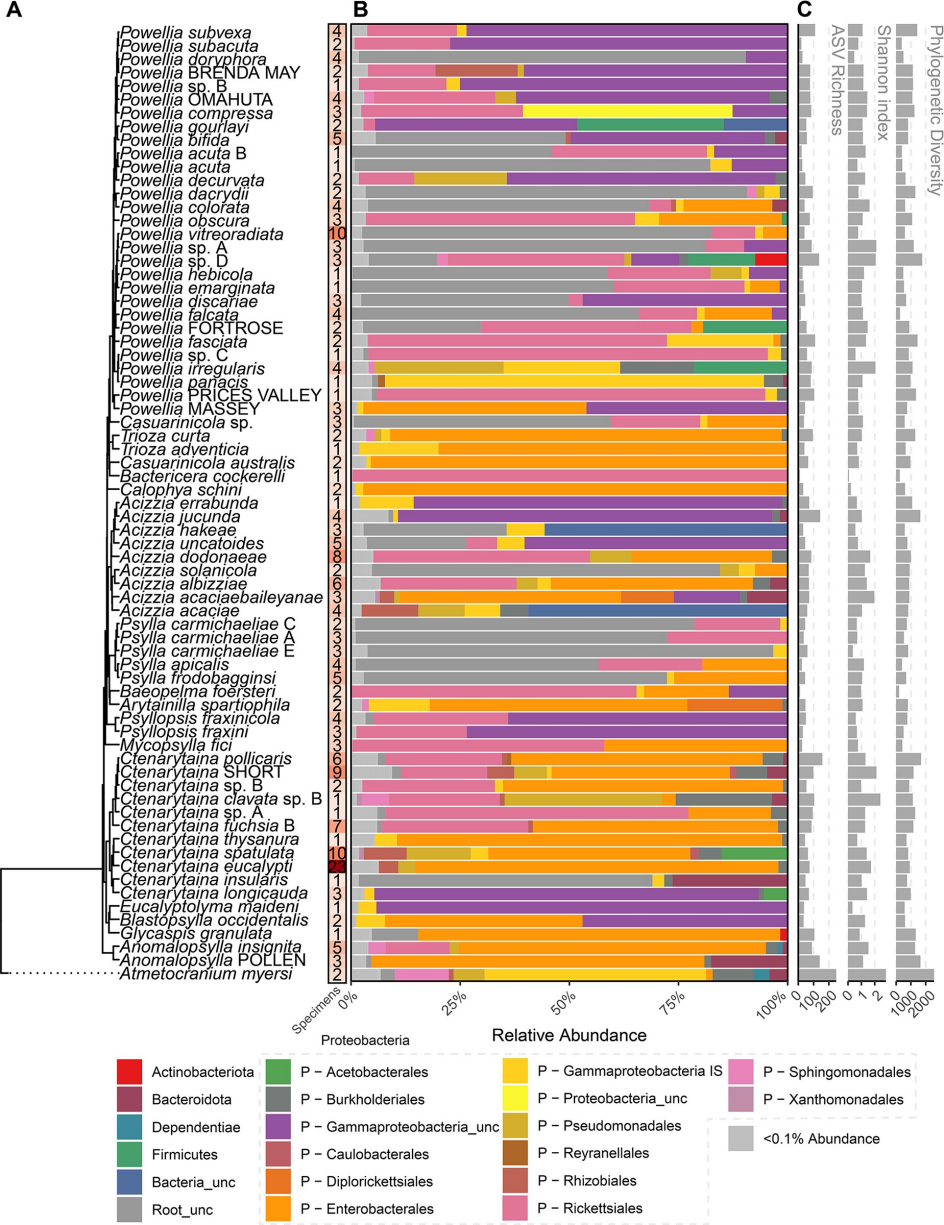
Overview of microbiome composition by psyllid species. A) Phylogeny of psyllid species, with N = number of specimens, shaded in red with increasing numbers. B) Overview of microbial Phyla recovered from each psyllid species, as well as orders within the dominant phylum Proteobacteria (P). Unc: Unclassified below this rank. IS: *Incertae sedis* C) α-diversity of the microbiome from each psyllid species. Following the current SILVA taxonomy release 138, *Ca*. Carsonella is placed within unclassified Gammaproteobacteria.

Once the phylogenetic structure of the Aotearoa New Zealand psyllids was inferred, a 16S metabarcoding analysis was conducted to characterize the bacterial microbiome of these species. Bacterial 16S sequences were generated from 277 specimens belonging to 246 individual insects (S2 Table), encompassing 75 psyllid species across 202 populations collected in Aotearoa New Zealand and Australia. Following quality filtering a total of 10,893,478 sequence reads were retained from 256 specimens of 75 taxa (mean  =  42,553±2,322; range  =  1,352:212,875). Sequences consisted of 3,763 unique amplicon sequence variants (ASVs) (mean  = 69.2±2.84; range  = 8:279) from 25 distinct bacterial phyla and 337 unique genera (Figs 2 and S1B). In addition, there were 657 ASVs that could not be assigned to any bacterial phyla using the IDTAXA algorithm and the SILVA v138 references database. However, they were retained for further analysis as supplementary BLAST searches revealed similarities between some unclassified ASVs and uncultured secondary symbiont sequences on the NCBI GenBank nucleotide database. Statistically significant differences were found between psyllid species for ASV richness (ANOVA; F_(76, 179)_ = 1.9, p < .001), Shannon’s index (ANOVA; F_(76, 179)_ = 2, p < .001), and phylogenetic diversity (ANOVA; F_(76, 179)_ = 2.01, p < .001). When all samples were rarefied to the sequencing depth of the lowest sample (1,352 reads) this pattern remained. The most abundant bacterial phylum was Proteobacteria accounting for 79% of the reads, followed by Bacteroidota (2.2%), Firmicutes (0.96%), and Actinobacteriota (0.3%). However, ASVs that could not be classified to phyla also accounted for 16% of the reads. The most abundant genus was *Wolbachia* within the proteobacterium order Rickettsiales, accounting for 16% of the total reads. While *Carsonella* of the Gammaproteobacteria_unclassified order was the most prevalent bacterial species in the dataset (detected in 243 of 254 specimens), it was generally present at a low relative abundance (5.8% of total reads, mean 1.6±0.26%). In particular *Ca*. Carsonella was not recorded in seven out of eight samples of *Anomalopsylla* and showed very low levels in the remaining specimen (8 reads). Additionally, *Carsonella* was not recorded in seven out of eight samples of *Psyllopsis*, and in a single specimen of *Glycaspis granulata* and *Psylla frodobagginsi*.

A diverse collection of *Enterobacterales* (order Gammaproteobacteria) with similarities to symbionts from other insects [24,98] dominated the dataset (Fig 2). Of this group, the two families with the highest number of reads were Morganellaceae, which includes *Arsenophonus* (2.8% of total reads) and *Hamiltonella* (1.3%), plus Pectobacteriaceae, which includes *Sodalis* (7.2%). *Arsenophonus* was found consistently amongst specimens within several psyllid species, being all four specimens of *Anomalopsylla* “Pollen Island”, the only specimen of *Casuarinicola australis* collected in Aotearoa New Zealand, all specimens of *Ctenarytaina eucalypti*, the only *Glycaspis granulata* specimen sequenced, all four specimens of *Powellia falcata* and all three specimens of the undescribed triozid species. *Sodalis* was recorded in two (out of three) specimens of *Acizzia acaciaebaileyanae* collected in Australia, all specimens of *Blastopsylla occidentalis* (N = 2) and 24 specimens of *Ctenarytaina eucalypti*. However, neither *Arsenophonus* nor *Sodalis* were recorded in specimens where infections of *Wolbachia* were detected, inferring a negative correlation/relationship between these two bacteria and *Wolbachia*, independent of psyllid species. Other detected Enterobacteriales included the plant and/or insect pathogen containing families of the Yersinaceae, detected in *Powellia*, *Ctenarytaina* and *Acizzia*, and Erwinaceae, detected in *Powellia*, *Ctenarytaina*, *Acizzia*, as well as one specimen of *Blastopsylla* and one of *Atmetocranium*. Furthermore, the inclusion in this dataset of psyllid species adventive to Aotearoa New Zealand revealed the same symbionts that have been recorded elsewhere for those same psyllid species, as was the case of the Enterobacteriaceae of *Mycopsylla fici* [99] and *Calophya schini* [98].

Besides Enterobacterales, other potential Proteobacteria symbionts included *Pseudomonas* (order Pseudomonadales) and Comamonadaceae (order Burkholderiales) ASVs present across the dataset (*Pseudomonas*; 2.6% of total reads, Comamonadaceae; 0.2% of total reads), with widespread occurrence across all genera in low read numbers for each sample (*Pseudomonas*; mean 1.72±0.32% across 62 specimens, Comamonadaceae; mean 0.12±0.01% across 60 specimens). Similarly, the other bacterial genera present as low abundance ASVs were represented across the bulk of the dataset. While a few bacterial genera were present in low read numbers across many species, including *Mycoplasma*, *Hymenobacter*, *Corynebacterium*, *Reyranella*, and *Novosphingobium*, they did not show any pattern of localization. Beyond the Proteobacteria, *Candidatus* Cardinium (order Sphingobacteriales) known for a similar reproductive association in insects as *Wolbachia* was detected (0.5% of total reads), but only in the psyllid specimens of *Anomalopsylla insignita*.

#### 3.2.2 β-diversity and phylosymbiosis: How the microbiome diversity is shaped by the insects phylogenetics

**Fig 3:**
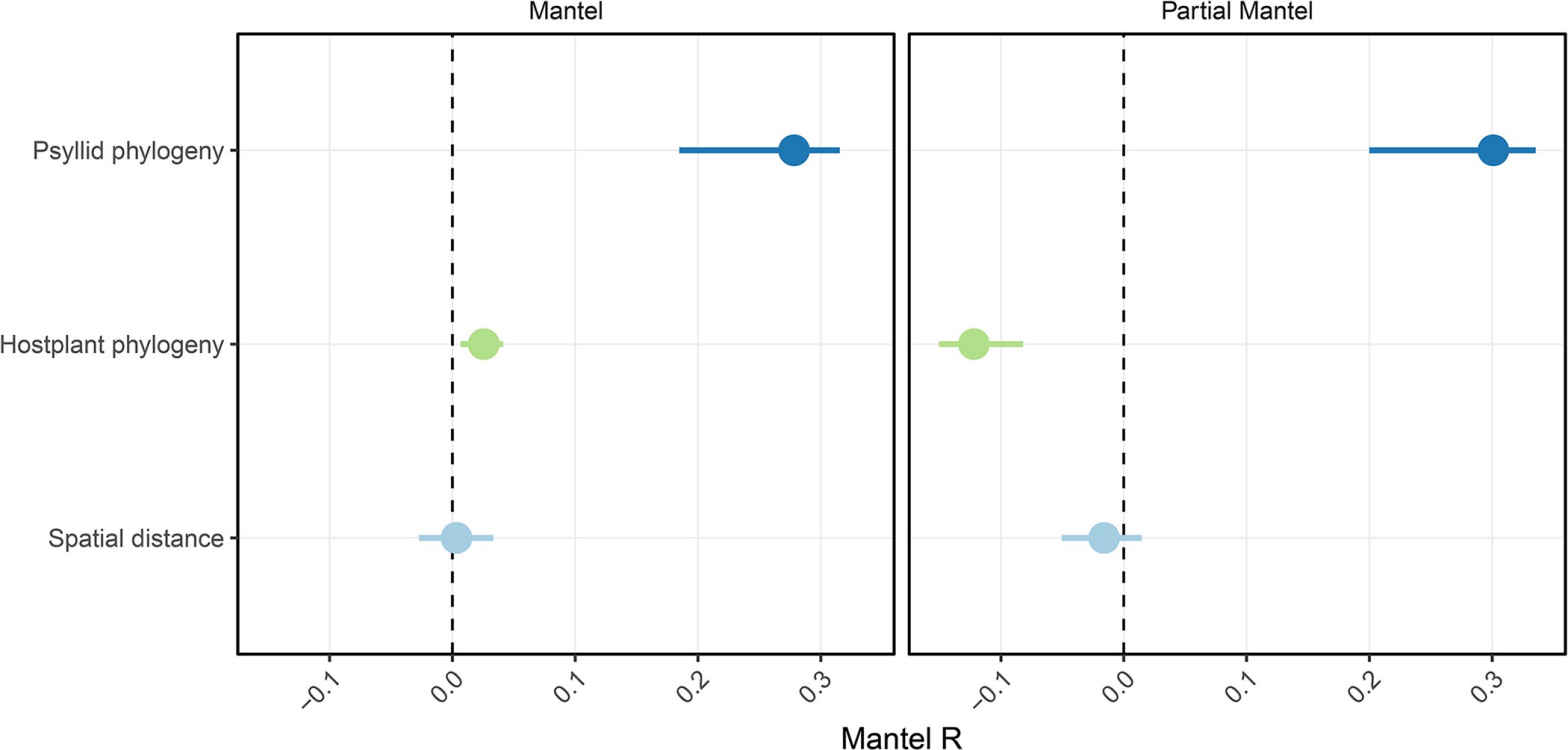
Phylosymbiosis. Results of Mantel tests (**left**), Partial Mantel tests (**right**), with 95% confidence intervals displayed.

To test the primary hypothesis, we used multivariate testing to find that the taxonomic identity of the psyllid host was a significant driver of compositional differences (bacterial ASV presence and abundance) between microbiomes (PERMANOVA; F_(56,179)_ = 1.48, R^2^ = .26, p < .001). However, significant differences in dispersion were also found between psyllid species, (PERMDISP; F_(74,181)_ = 5.96, p < .001) suggesting that the differences in microbiome composition between psyllids may be partly due to some species having more variation in bacterial taxa compared to others [100]. PCoA plots of pairwise Aitchison distances supported this analysis, showing substantial dispersion between many psyllids from the same genus across both PC1 and PC2 (S2A, S2B and S2C Fig). The phylogenetic distance between psyllid species was significantly correlated with differences in microbiome β-diversity (Mantel’s r = .27, p < .001), indicating that greater phylogenetic divergence between psyllid hosts had led to less similar microbiome compositions (Fig 3A). This significant positive correlation remained when host plant phylogenetic distance and spatial distance were controlled for (Partial Mantel’s r = .3, p < .001). However, this could not clarify if the host plant associations were playing a major role in the psyllid microbiome composition as well, due to the fact that phylogenetically distinct psyllids tend to be on phylogenetically distinct host plants (Fig 1).

The taxonomic identity of the host plants of each psyllid also explained a significant amount of variance (PERMANOVA; F_(55,198)_ = 1.45 R^2^ = .26, p < .001) and dispersion in microbiome composition (PERMDISP; F_(55,200)_ = 5.63, p < 0.01), but psyllids collected from phylogenetically similar host plants did not harbour more similar microbiomes (Mantel’s r = .04, p = .19). In fact, when psyllid phylogenetic distance and spatial distance were controlled for, a slight negative correlation was found between microbiome β-diversity and host plant phylogenetic distance (Partial Mantel’s r = -0.12, p = .004), suggesting that more phylogenetically related host plants harbour psyllids with less similar microbiomes (Fig 3B). On the other hand, the relationship between β-diversity and spatial distance was not significant when psyllid and host plant phylogenetic distances were controlled for (Partial Mantel’s r = .016 p = .78) (Fig 3B). This indicated that the detected patterns of phylosymbiosis between the psyllids phylogenetics and their microbiome were not confounded by these other covariates, i.e., host plant and geographic distribution. For psyllid specimens of different species which were collected from the same single host plant at the same time point, such as *Psyllopsis fraxini* and *Psyllopsis fraxinicola* collected from *Fraxinus excelsior*, or *Psylla apicalis* and *Psylla frodobagginsi* collected from *Sophora microphylla*, there were no significant differences found between their microbiomes (*Psyllopsis fraxini*/ *fraxinicola* R^2^ = .29, p > .5; *Psylla apicalis*/*frodobagginsi* R^2^ = .44, p > .5). While this can be considered a preliminary analysis due to the limited number of samples, the results suggest that there is some sharing of host-acquired microbes between closely related species when they are present on the same host plant. However, only 37 of 175 ASVs were shared between the *Psyllopsis fraxini* and *Psyllopsis fraxinicola* specimens, and only 24 of 101 shared between the *Psylla apicalis* and *Psylla frodobagginsi*, which suggests that these host acquired microbes represent a small portion of the microbiome.

To test our secondary hypothesis that factors beyond the co-phylogenetic signal between insect hosts and their symbionts may be important in shaping the bacterial microbiome, and to determine if the correlation between β-diversity and phylogenetic distance was not driven only by the well-known symbiont taxa, Partial Mantel tests were repeated excluding the bacterial class Gammaproteobacteria which includes both the primary and known co-primary symbionts [23]. Within this subset of data, a significant, correlation was still found between β-diversity and psyllid phylogenetic distance (Partial Mantel’s r = .32, p < .001), and similarly the slight negative correlation with host plant phylogenetic distance remained (Partial Mantel’s r = -.14, p < .001). The relationship between β-diversity and geographical distance remained insignificant (p > 0.5).

#### 3.2.3 Co-phylogeny

**Fig 4:**
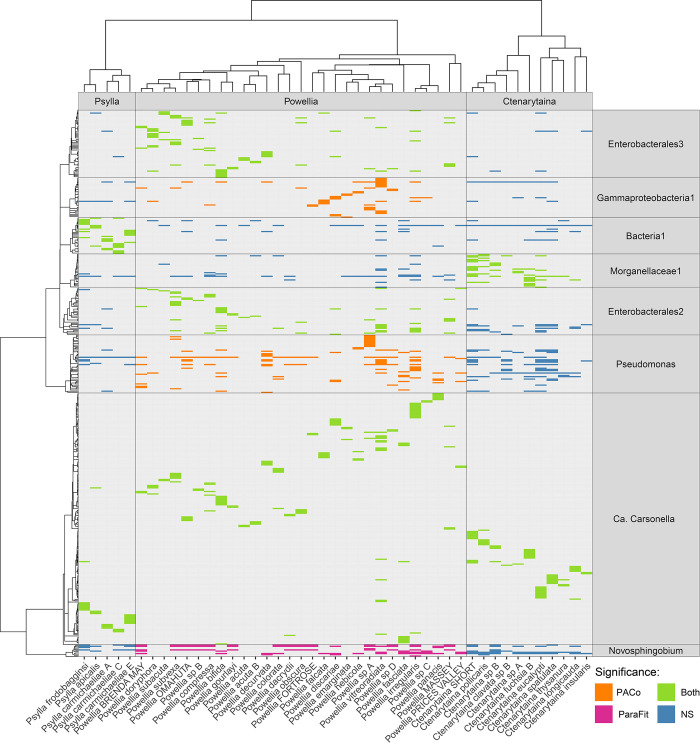
Phylogenetic congruence between *Psylla*, *Powellia* and *Ctenarytaina* species and the core OTUs that were found to show significant co-phylogenetic signal with PACo or Parafit.

With the psyllids’ phylogenetics playing a major role in shaping the microbiome composition, we further investigated the co-evolution between the three major psyllid genera in Aotearoa New Zealand (Fig 4) and their core microbiome. The core microbiome was designated as 43 lineages that were found to be present in >25% of species in at least one of the main genera, with more than five unique variants (S3 Fig). As expected, significant evidence for co-evolution with *Carsonella* was found across all three major psyllid genera (*Psylla*; PACo m^2^_xy_ = .36, ParaFitGlobal = 37.15, *Powellia* PACo m^2^_xy_ = .42, ParaFitGlobal = 1509.26, *Ctenarytaina*; PACo m^2^_xy_ = .26, ParaFitGlobal = 328.50, all p < .001), demonstrating that this approach is suitable for identifying known vertically inherited symbionts (Figs 4 and S4). For *Powellia*, significant co-phylogenetic signal was found with both PACO and Parafit for two lineages other than *Carsonella*, the Enterobacterales clade 2 (PACo m^2^_xy_ = .23, ParaFitGlobal = 309.9, both p < .001) (S5 Fig), and Enterobacterales clade 3 lineages (PACo m^2^_xy_ = .54, ParaFitGlobal = 398.4, both p < .001) (S6 Fig). Both these lineages predominantly contained unclassified Gammaproteobacteria ASVs, as well as others that were assigned to higher ranks only (S5 and S6 Figs). In addition, two bacterial lineages were found to show significant co-phylogenetic signal with *Powellia* with PACo but not ParaFit, namely the Gammaproteobacteria clade 1 (PACo m^2^_xy_ = .59 p < .001, ParaFitGlobal = 55.48 p > 0.05), and *Pseudomonas*-like clade (PACo m^2^_xy_ = .84 p < .001, ParaFitGlobal = 206.81 p > 0.05), while the *Novosphingobium*-like clade was found to show significant co-phylogenetic signal with ParaFit but not PACo (PACo m^2^_xy_ = .93 p > 0.05, ParaFitGlobal = 86.7 p = .001) (Fig 4). The Gammaproteobacteria clade 1 contained several unclassified Gammaproteobacteria ASVs and other ASVs classified to higher ranks only (S9 Fig), while the *Pseudomonas*-like clade predominately contained *Pseudomonas* and Pseudomonadaceae ASVs (S11 Fig). The *Novosphingobium*-like clade on the other hand contained only ASVs classified to *Novosphingobium* or Sphingomonadaceae (S10 Fig). For Psylla, apart from Carsonella, significant co-phylogenetic signal was found for only one other lineage, the Unclassified bacteria 1 clade (PACo m^2^_xy_ = .65, ParaFitGlobal = 111.15, both p < .001, Fig 4), which contained 30 ASVs that could not be assigned to lower-level taxonomy, of which 28 were present across the genus *Psylla* (S8 Fig). Despite these ASVs not being classifiable to lower taxonomic ranks with IDTAXA, further BLASTn searches against GenBank found the closest matches to be putative Enterobacteriaceae endosymbionts from other psyllid species, suggesting this lineage may contain microbes with secondary symbiont functions in *Psylla*. For *Ctenarytaina*, significant co-phylogenetic signal was found with both approaches for Morganellaceae clade 1 (PACo m^2^_xy_ = .64 p < .001, ParaFitGlobal = 40.23 p = 0.23, Fig 4), predominately containing ASVs classified to Morganellaceae or Enterobacterales (S7 Fig).

## 4. Discussion

### 4.1. Factors influencing the microbiome composition of the psyllids of Aotearoa New Zealand

#### 4.1.1 Phylosymbiosis and other evidence of co-phylogeny

The co-evolutionary relationship between sap feeding insects and their nutritional symbionts is one of the best-known examples of phylosymbiosis [12], which is tested for by determining if insect species and host plants can be distinguished by their microbiome and if genetically more similar insect species harbour more similar microbiomes [32]. Accordingly, the primary hypothesis of this study, that the psyllid phylogenetics is the main factor influencing their microbiome composition, was supported by both the PERMANOVA analysis and the Mantel and Partial Mantel tests. This result, albeit not surprising, highlighted a novel case of phylosymbiosis, which had never been formally reported for psyllids. Additionally, we detected strong signals of co-evolution with *Carsonella*, and other putative primary symbionts, as well as known secondary symbionts within the Enterobacteraceae [10,17,18,23,26]. A clear example of a psyllid-microbiome co-evolutionary relationship was observed in the closely related *Psyllopsis fraxini* and *P*. *fraxinicola* found feeding on the same host plant species, *Fraxinus excelsior*, including on the same individual plant on three occasions. Independent of the geographic collection location, *P*. *fraxini* showed a high abundance of an Enterobacteriaceae sequence that was not found in any of the *P*. *fraxinicola* samples, even those collected from the same individual tree. The Enterobacteriaceae strain was identical across all three populations of *P*. *fraxini*, suggesting this is an instance where vertical transmission of an Enterobacteriaceae symbiont within its psyllid host has occurred. The absence of this Enterobacteriaceae strain from the second, genetically similar psyllid species (*P*. *fraxinicola*) present on the same host plant, highlights how these symbionts are not horizontally transmitted even between closely related psyllid species feeding on the same individual plant.

To test our secondary hypothesis, that factors beyond bacterial lineages are important in shaping the microbiome composition, all known psyllid symbionts were removed from the total dataset which was then reconsidered for host plant and geographic distribution associations. The finding was that when all Gammaproteobacteria, including *Carsonella* and secondary symbionts belonging to the Enterobacterales (e.g., *Sodalis*) were removed, significant signals of phylosymbiosis remained. Other bacterial genera that showed significant co-phylogenetic congruence with the psyllid host include *Pseudomonas*, Morganellaceae and *Novosphingobium*. Elsewhere, these bacteria have been found to have a remarkable phenotypic diversity, including environmental bacteria from water and soil habitats, sometimes capable of degrading aromatic compounds (i.e., *Novosphingobium–*[101]). Most of these bacteria have been found previously in psyllid microbiomes [25,102]. The fact that they were not only recorded here but also found to have a significant co-phylogenetic signal, may suggest that they are part of a “core microbiome” associated with psyllids, and not merely linked to the environment. This suggests that psyllid microbiome composition has a strong phylosymbiotic signal that it is not limited to the few primary and secondary bacteria but may have deeper roots in bacterial lineages with previously unknown symbiotic roles. These bacteria are hypothesised here to be vertically transmitted and further studies aiming to determine psyllid-bacteria co-evolutionary histories should focus on these lineages to further improve our understanding of transmission mechanisms.

Here, we also tested the co-phylogenetic signals for bacteria that had previously been considered “non symbiotic” within psyllid lineages that have different host plant associations. When compared for each of the three major psyllid genera in Aotearoa New Zealand (Fig 4), significant evidence was found for co-evolution within *Psylla* (limited host plant associations), *Ctenarytaina* (few host plant associations) and *Powellia* (multiple host plant associations). *Psylla* showed a significant co-phylogenetic signal with an unknown bacterial clade, supported by both PACo and ParaFit, suggesting this clade could play a symbiotic role and be potentially vertically transmitted. *Ctenarytaina* showed a significant co-phylogenetic signal with bacteria from the Morganellaceae clade, while signals from all other clades appeared to be non-significant. On the other hand, however, *Powellia* appeared to have the highest number of significant co-phylogenetic signals with bacteria within the clades Enterobacterales, Gammaproteobacteria, *Pseudomonas* and *Novosphingobium*. In the case of the *Powellia*, further examination with a focus on the bacteria presenting significant PACo and ParaFit signals is recommended to explore if any bacterial lineage has contributed evolutionary advantage to the psyllids, perhaps to enable species to switch plant hosts. Ultimately, these significant co-phylogenetic signals suggest some of these bacteria could be vertically transmitted and play a symbiotic role within their psyllid hosts.

However, it is important to consider that patterns of phylosymbiosis are not necessarily driven by co-evolution or vertical transmission. They could instead be driven by host filtering whereby closely related psyllids share similar phenotypic traits (e.g., diet, gut pH, gut morphology) which may favor similar preadapted bacteria from the environment [103]. Further studies will have to focus on bacterial biosynthesis pathways to determine if their presence in the psyllid can be considered a symbiosis whereby specific amino acids are produced, or if the bacteria play a role in the protection of the insects.

#### 4.1.2 Bacterial diversity

*Carsonella* is the known obligate primary symbiont of all psyllids and its role as provider of essential amino acids has been widely studied ([104] and references therein). Here, *Carsonella* was generally recorded at low relative abundance, as observed in other studies (e.g., [25,102]), but still showed a very strong phylogenetic association with their psyllid hosts having genetically different haplotypes for each psyllid species. While the limited resolution for deeper phylogenetic nodes provided by the short bacterial 16S gene sequences used here likely resulted in the phylogenetic trees of psyllids and *Carsonella* not overlapping more closely, horizontal gene transfer may have also played a part. A longer fragment of the same gene and/or additional genes could potentially improve this. Interestingly, *Carsonella* was not detected in four of the psyllid species, most likely due to insufficient sequencing depth or mismatch of the generic 16S primers (as shown elsewhere [25]), rather than actual absence of the obligate primary symbiont [17,20]. However, in all specimens of *Anomalopsylla*, along with no detectable *Carsonella*, either *Cardinium* or *Hamiltonella* were recorded in higher abundance compared to other species where *Carsonella* was detected. Further studies are needed to examine what metabolic pathways are provided by *Cardinium* and *Hamiltonella* to determine if they have a potential substitute role as co-primary symbionts to provide the psyllid with amino acids. The complete absence of *Carsonella* has not been proven for any psyllid, however, other genomic analyses have shown that there can be an absence of *Carsonella* genes involved in the amino acid metabolic pathways in some psyllid species [22]. This may therefore indicate that at least part of the amino acid provision may be compensated for by native host genes or horizontal gene transfer from bacteria to the host [27] and there could be a co-primary role played by *Hamiltonella* and *Cardinium* in *Anomalopsylla* species.

Two *Wolbachia* strains occurred often at a high levels across individuals of several species, genera, and families although their distribution (presence/absence) as well as the relative quantity of reads was variable within the same species, and even within the same population. The small genetic distance between these strains (always <2%, both from Aotearoa New Zealand and Australia specimens) suggests that *Wolbachia* does not have a long history of co-evolution with its psyllid hosts. This is in accordance with what has been observed for *Wolbachia* in other psyllids, with strains that are often genetically identical across the world [25,99,102,105]. Elsewhere, *Wolbachia* has been hypothesized to play an endosymbiotic role for psyllids (e.g., [25]), with some studies suggesting its presence may be associated with cytoplasmic incompatibilities between different psyllid haplotypes [106] or positively correlated with pathogen abundance [107]. In other insects, such as fruit flies (Diptera: Tephritidae), *Wolbachia* has been shown to be transmitted by associated parasitoid wasps as a means of horizontal gene transfer [108]. This mechanism of transmission of genetic material suggests that its presence within a microbiome can arise from repeated infections, but it remains unclear what role is played by this bacterium within the psyllid microbiome and whether this is a case of symbiosis of not. *Wolbachia* is known to manipulate reproduction and induce other fitness benefits under times of nutrient stress in insects [25,109]. It has also been shown to be a bacteriocyte-associated mutualist in white flies [109], but this has never been confirmed for psyllids. Here, we detected *Wolbachia* at high abundance where no other Enterobacteriaceae symbiont (i.e., *Sodalis*, *Arsenophonus*) was found.

Beside *Carsonella* and *Wolbachia*, the most prevalent group of bacteria was Enterobacterales. These have been considered potential secondary symbionts since they have been recorded elsewhere in psyllids [18,23,26]. Both *Arsenophonus* and *Sodalis* were usually present at high read numbers in very localized groups of psyllids, and neither were recorded where infections of *Wolbachia* were detected.

Enterobacteriaceae symbionts have previously been characterized in *Calophya schini* from Brazil [98] and *Mycopsylla fici* from Australia [99], two non-native psyllid species that have been introduced to Aotearoa New Zealand. When these previously published symbiont sequences were compared to the Enterobacteriaceae ASVs identified in this study, identical sequences were found, highlighting the strong conservation of Enterobacteriaceae secondary symbionts across distinct geographic regions. Other Enterobacterales detected here include Yersinaceae and Erwinaceae, families known to contain plant pathogens as well as insect symbionts [110]. Erwinaceae strains have also been recorded in psyllids from Australia [25], but they were considered to have possible transient microbial associations. *Reyranella* (Rhodospirillales) was generally found in very low read numbers across all genera, while *Mycoplasma* (Mycoplasmatales) was much more restricted within *Powellia colorata*, as well as various Aotearoa New Zealand native *Ctenarytaina* and in *Acizzia*.

Finally, *Pseudomonas* (Pseudomonadales) was detected in more than 3% of total reads across most of the psyllid species, consistent with the high relative abundance in *Trioza* and *Pauropsylla* species elsewhere [25]. Here we were able to demonstrate a high co-phylogenetic signal (see below), indicating that *Pseudomonas* may have a more important role for the genus *Powellia* than previously hypothesized as a transient microbial association [25]. More specific PCR primers and a focus on biosynthetic pathways may be useful in further studies of *Pseudomonas* function within the psyllid microbiome.

### 4.2. Microbiome-host plant relationships

Understanding the relationship of insect symbionts to host plant adaptation is an emerging field [35,104]. Recently, McLean and colleagues found a strong signal of aphid evolution with microbiota but only a weak signal derived from the host plant [111]. Another work [112] observed a significant correlation between aphid phylogeny and microbial community composition but no association with aphid hosts and a lesser signal between microbiome and host plant phylogeny. The results obtained here are in general agreement with those studies.

The molecular data generated in our study reveals the native psyllid species diversity observed in Aotearoa New Zealand today is the result of evolutionary radiations that happened within the country. The contemporary psyllid diversity originated from an initial six ancestral lineages, each of these diverged to produce endemic species within *Atmetocranium*, *Anomalopsylla*, *Ctenarytaina*, *Psylla* and *Trioza*, as well as the mostly endemic genus *Powellia*. When considering the host plant associations across the three main native psyllid genera—*Psylla*, *Ctenarytaina* and *Powellia—*two main patterns can be observed. The first one is well represented by the two psyllid genera *Ctenarytaina* and *Psylla*, which are associated with only one or a few host plant families, supporting the strong host plant specificity of psyllids [3] and being quite consistent with observations of psyllids worldwide (e.g., [6]). However, a different pattern can be observed for *Powellia*, which is recorded on 11 host plant families across the 30 psyllid species included here (Fig 1). This pattern has been widely observed for triozid species, especially within the genus *Trioza* from which *Powellia* has been recently separated [39]. Within the genus *Trioza*, 346 species are associated with 154 plant genera and 59 families [6] but this psyllid-plant association has been hypothesized to be due to the taxonomically paraphyletic nature of that particular genus [6,7]. While polyphyly was observed here for the fraction of the *Trioza* represented (Fig 1), we also confirmed the monophyly of *Powellia*, strongly suggesting that underlying polyphyly is not the cause of the diverse host plant associations seen across this genus. This conclusion suggests that *Powellia* is (or has been) potentially more prone to host switching, as has been highlighted for the monophyletic *Diaphorina* [1,9], for which more than 70 species have been found associated with 42 genera and 22 families [1].

Psyllid microbiome composition, even when removing the known primary and secondary symbionts, is more strongly correlated with the insects’ phylogenetic relationships as opposed to that of the host plants. However, it is difficult to disentangle the role of host plants and of host insect phylogenetics where strong insect-host plant specificity occurs, such as in psyllids. Indeed, the strong species-specific association between psyllids and their hosts can be reflected in the psyllids’ microbiome composition, with psyllid species that feed on different plants showing different microbial diversities which is linked to the psyllids phylogenetic distance rather than that of the host plants. While undoubtedly several bacterial species enable a psyllid species to feed on a specific plant, the general microbiome composition of psyllids appears to have co-evolved with the insects and not with the associated host plant. For example, here the microbiome of *Powellia* appears to be more similar to that of *Psylla* as opposed to that of *Ctenarytaina* and is explained by the closer phylogenetic relationships between Psyllidae and Triozidae than between any of these two families and Aphalaridae. While at a genus level some psyllids may show a wide host range, for example in the case of the genus *Powellia*, at a species level they remain host specific and feed on a single plant. Furthermore, the associations of *Powellia* species with a wider number of host plants could not be characterized by the presence of specific bacterial taxa (i.e., found in *Powellia* and nowhere else). This suggests that if specific bacterial groups had been responsible for ancestral host switches of *Powellia* species, enabling the present-day species to feed on more than 11 plant families, such bacteria could not currently be detected. Although, this could be due to insufficient resolution in our metabarcoding approach. With longer amplicons/full genes it may be possible to further differentiate species that we currently could only ascribe to the same genus. The presence of such ancestral bacteria responsible for host switches could be linked to transient infections caused by horizontally transmitted bacteria that an ancestral psyllid could have acquired from plants they had casually come into contact with. A transient association between insects and bacteria has been reported before [113] and has the potential to translate to a more stable insect-bacteria relationship when the microbiome mediates insect-plant associations (i.e., enabling an insect to feed on a plant [114]). Indeed, psyllids can be found on plants other than their host plant, where they may obtain nutrition (food plants) and shelter (shelter plants), despite these not supporting the complete immature-to-adult life cycle (therefore not being considered as “host plants”–[2,115]). Such casual psyllid-plant interactions may have led to transient infections, plausibly similar to those observed here for *Wolbachia*, which could have enabled the ancestral psyllids to feed on different plants until a new essential amino acid metabolic pathway was facilitated by—or horizontally transmitted to—primary and secondary symbionts. This hypothesis is supported by recent studies suggesting that bacteria living on the surface of leaves may play a greater role in insect-host adaptation [114], and that transient symbiont associations may contribute to enabling a rapid host plant switch for sap-feeding insects [116]. Furthermore, in psyllids the same amino acids have been shown to be produced by secondary symbionts as well as the primary one, either by sharing the same biosynthesis capabilities, or by having complementary capabilities [23]. The association with bacteria producing additional amino acids could allow the psyllid to feed on a different host, as has been demonstrated for aphids [117,118]. If the genes responsible for the amino acid biosynthesis have since been provided by additional symbionts, the ancestral symbiont responsible for the host plant switch may not need to be present in the microbiome today. An example might be with the *Powellia* where no single bacterium can be currently identified as responsible for their host switches and the microbiome of these psyllids remains more strongly associated with the insects’ phylogenetics. Another hypothesis, however, can be linked to the possibility of some genes being horizontally transmitted without having to be carried by any specific bacterial genome. This has been widely documented by Sloan and colleagues [27], who presented horizontal gene transfer as a recurring mechanism driving co-evolution between insects and their bacterial symbionts. A more recent study on whiteflies even recorded an exceptional horizontal gene transfer event where the insect acquired a plant-derived gene [119]. If *Powellia*’s ability to associate with a wide range of host plant families was indeed the result of one or more horizontal gene transfer event(s), then no specific bacterial lineage could be pinpointed as the sole causal agent. On the other hand, widespread bacteria present across the microbiomes of different psyllid groups may carry different genes enabling them to feed on different plants or to synthesize different amino acids. In order to test these hypotheses, analysis of the metabolic biosynthesis pathways should be performed on psyllid symbionts, including those recorded here for the first time. Furthermore, full genome sequencing of the bacteria could help determine whether horizontally transmitted genes are present in certain bacterial strains and not in others.

## Supporting information

S1 Fig **A. Rarefaction curves.** Rarefaction curves showing sequencing depth for each sample. All samples below 1000 sequence reads were removed. **B. Bacterial abundance and prevalence.** Abundance and prevalence of different bacterial phyla, and unclassified ASVs, across the entire dataset after filtering. Each point coloured by taxonomic order.
(TIF)

S2 Fig **A. Bacterial community and psyllid phylogeny.** PCA plot of Aitchison distance between microbial communities coloured by psyllid phylogenetic cluster. **B. Bacterial community and plant phylogeny.** PCA plot of Aitchison distance between microbial communities coloured by host plant phylogenetic cluster. **C. Bacterial community and geographic distribution.** PCA plot of Aitchison distance between microbial communities coloured by cluster membership following UPGMA on great circle distance between collection locations.
(TIF)

S3 Fig Bacterial OTUs across psyllid genera. Heatmap of all core OTUs across the 3 main genera, coloured by whether significant co-phylogenetic signal was found with PACo or ParaFit.
(TIF)

S4 Fig Phylogenetic congruence between psyllid species and *Candidatus* Carsonella ASVs. Links and taxa coloured by whether significant co-phylogenetic signal was found with PACo or ParaFit. Due to the number of Ca. Carsonella ASVs, only a subset of labels is displayed for readability.
(TIF)

S5 Fig Phylogenetic congruence between psyllid species and Enterobacterales clade 2 ASVs. Links and taxa coloured by whether significant co-phylogenetic signal was found with PACo or ParaFit.
(TIF)

S6 Fig Phylogenetic congruence between psyllid species and Enterobacterales clade 3 ASVs. Links and taxa coloured by whether significant co-phylogenetic signal was found with PACo or ParaFit.
(TIF)

S7 Fig Phylogenetic congruence between psyllid species and Morganellaceae clade 1 ASVs. Links and taxa coloured by whether significant co-phylogenetic signal was found with PACo or ParaFit.
(TIF)

S8 Fig Phylogenetic congruence between psyllid species and Unclassified bacteria clade 1 ASVs. Links and taxa coloured by whether significant co-phylogenetic signal was found with PACo or ParaFit.
(TIF)

S9 Fig Phylogenetic congruence between psyllid species and Gammaproteobacteria clade 1 ASVs. Links and taxa coloured by whether significant co-phylogenetic signal was found with PACo or ParaFit.
(TIF)

S10 Fig Phylogenetic congruence between psyllid species and *Novosphingobium*-like ASVs. Links and taxa coloured by whether significant co-phylogenetic signal was found with PACo or ParaFit.
(TIF)

S11 Fig Phylogenetic congruence between psyllid species and *Pseudomonas*-like ASVs. Links and taxa coloured by whether significant co-phylogenetic signal was found with PACo or ParaFit.
(TIF)

S1 Table Psyllid samples used in this study for insect phylogenetic analysis. The table lists the species analysed and the family they belong to. Information on the endemicity to New Zealand of the species is reported for invasive (I), native (N), endemic (E) and non-present (NP) species. Collection location for the samples is provided together with the number of samples and populations analysed in this study. The number of DNA sequences used is reported together with accession numbers for the COI, EF-1α and 18S genes. Accession numbers in bold are for the sequences generated in this study.
(DOCX)

S2 Table Psyllid samples used in this study for bacterial 16S metabarcoding analysis. The table lists the samples analysed in this study and it lists information on their taxonomy (species, genus and family), populations ID, host plant, and collection location.
(XLSX)
