# Supplementary figures and images for: Insect phylogeny structures the bacterial communities in the microbiome of psyllids (Hemiptera: Psylloidea) in Aotearoa New Zealand
Source: PLoS One. 2023 May 15;18(5):e0285587. doi: 10.1371/journal.pone.0285587 (PMC10184942; doi:10.1371/journal.pone.0285587)

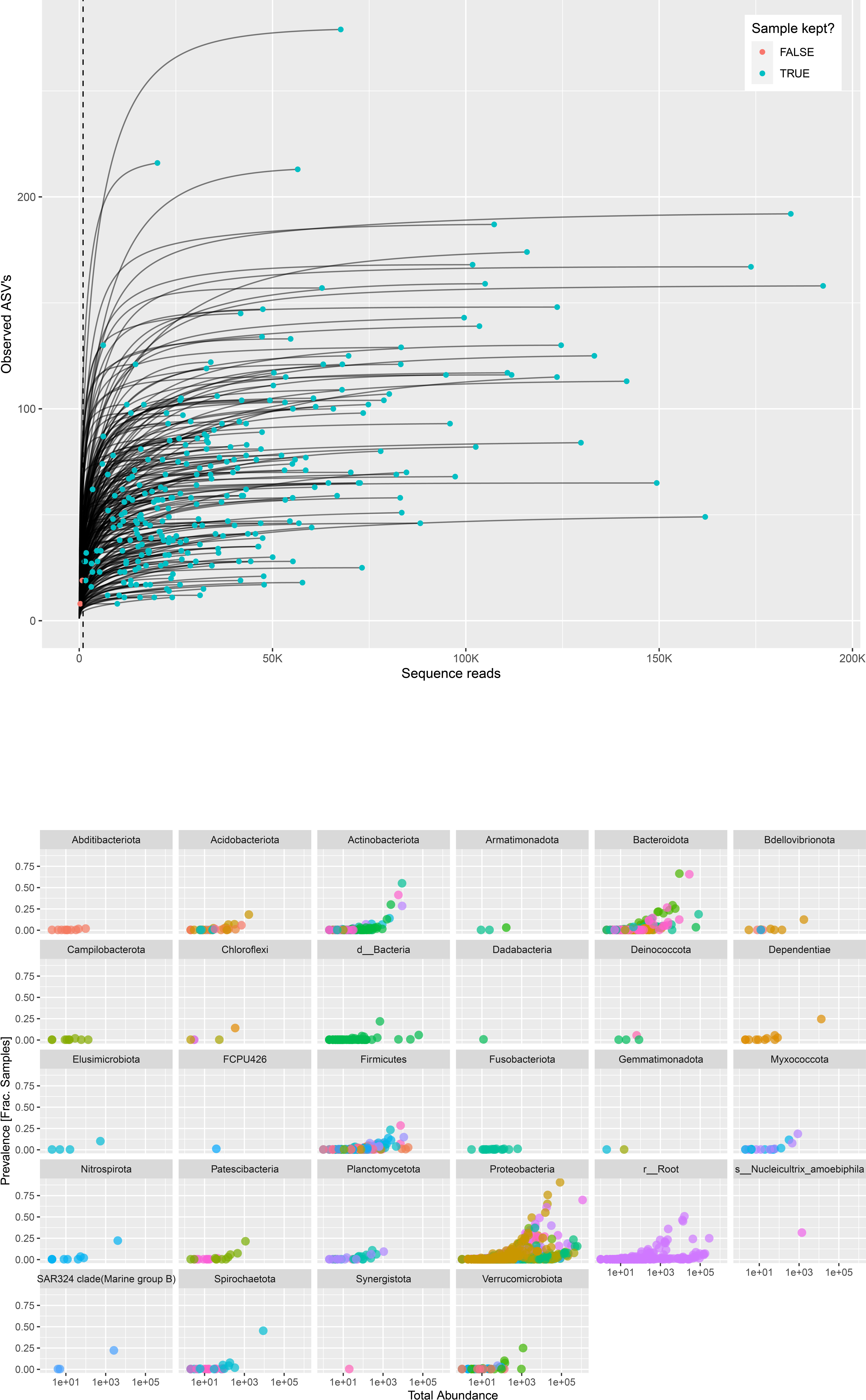

Supplement: S1 Fig — A. Rarefaction curves. Rarefaction curves showing sequencing depth for each sample. All samples below 1000 sequence reads were removed. B. Bacterial abundance and prevalence. Abundance and prevalence of different bacterial phyla, and unclassified ASVs, across the entire dataset after filtering. Each point coloured by taxonomic order. (TIF) [file pone.0285587.s001.TIF]

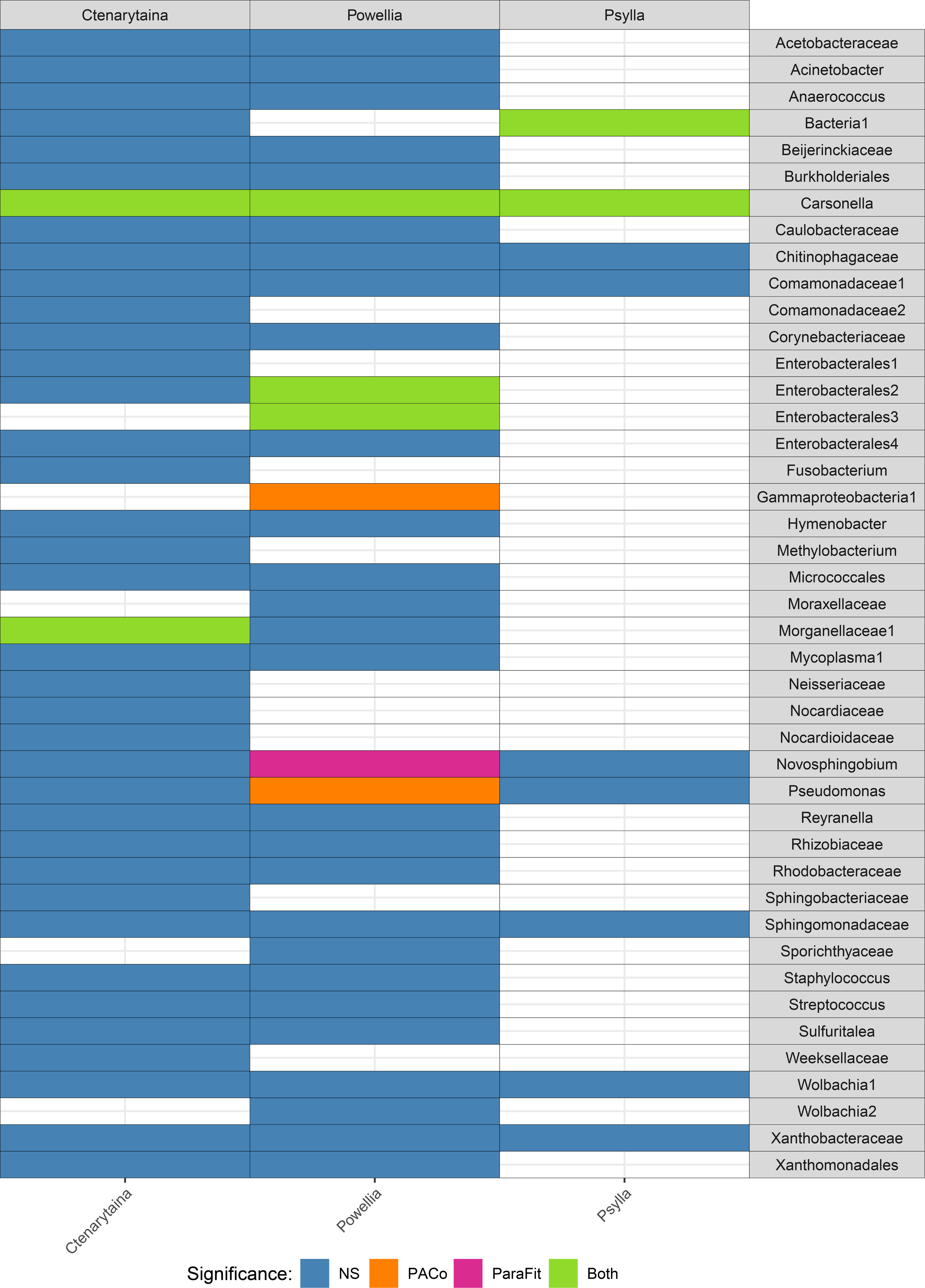

Supplement: S3 Fig — Heatmap of all core OTUs across the 3 main genera, coloured by whether significant co-phylogenetic signal was found with PACo or ParaFit. (TIF) [file pone.0285587.s003.TIF]

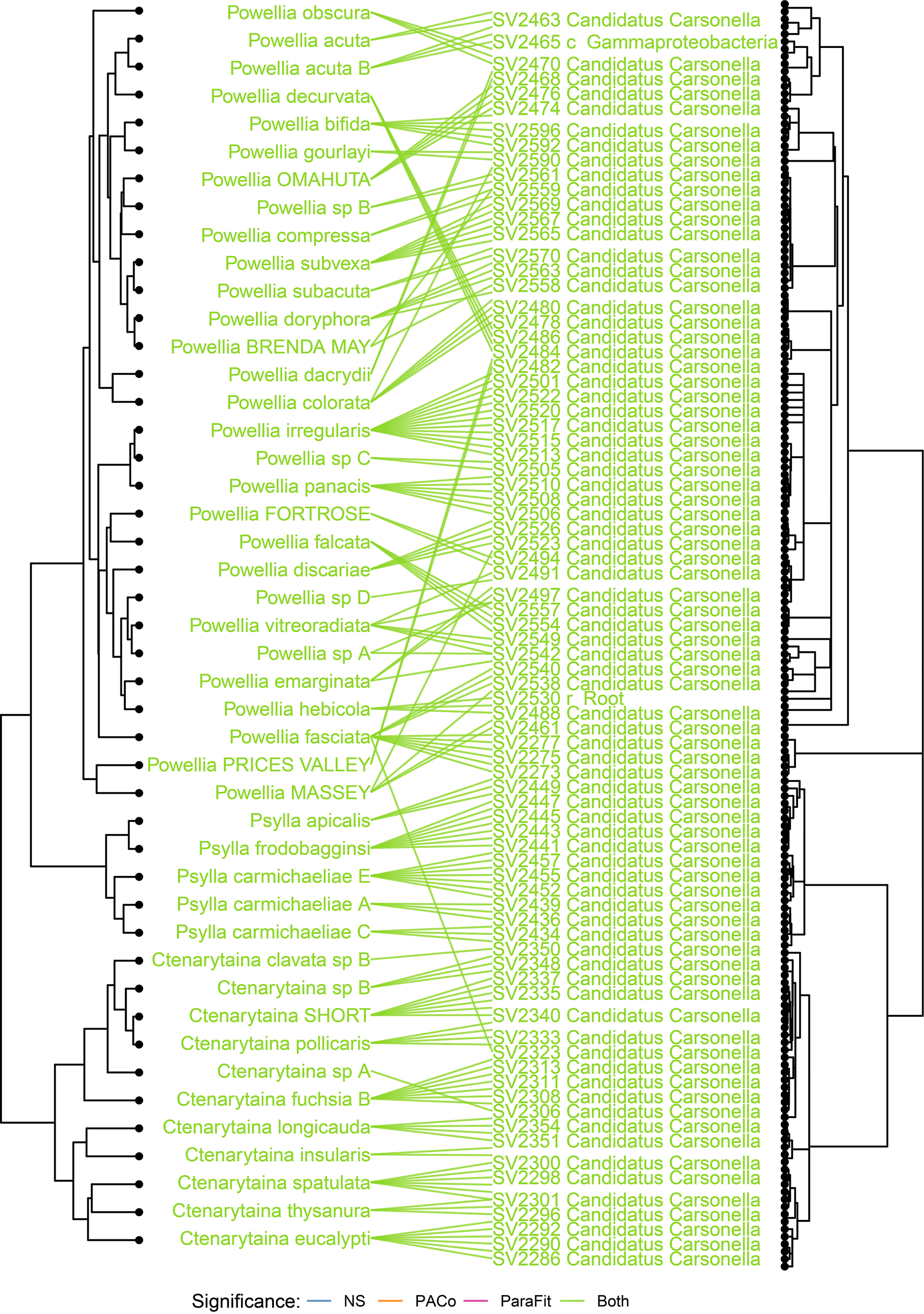

Supplement: S4 Fig — Links and taxa coloured by whether significant co-phylogenetic signal was found with PACo or ParaFit. Due to the number of Ca. Carsonella ASVs, only a subset of labels is displayed for readability. (TIF) [file pone.0285587.s004.TIF]

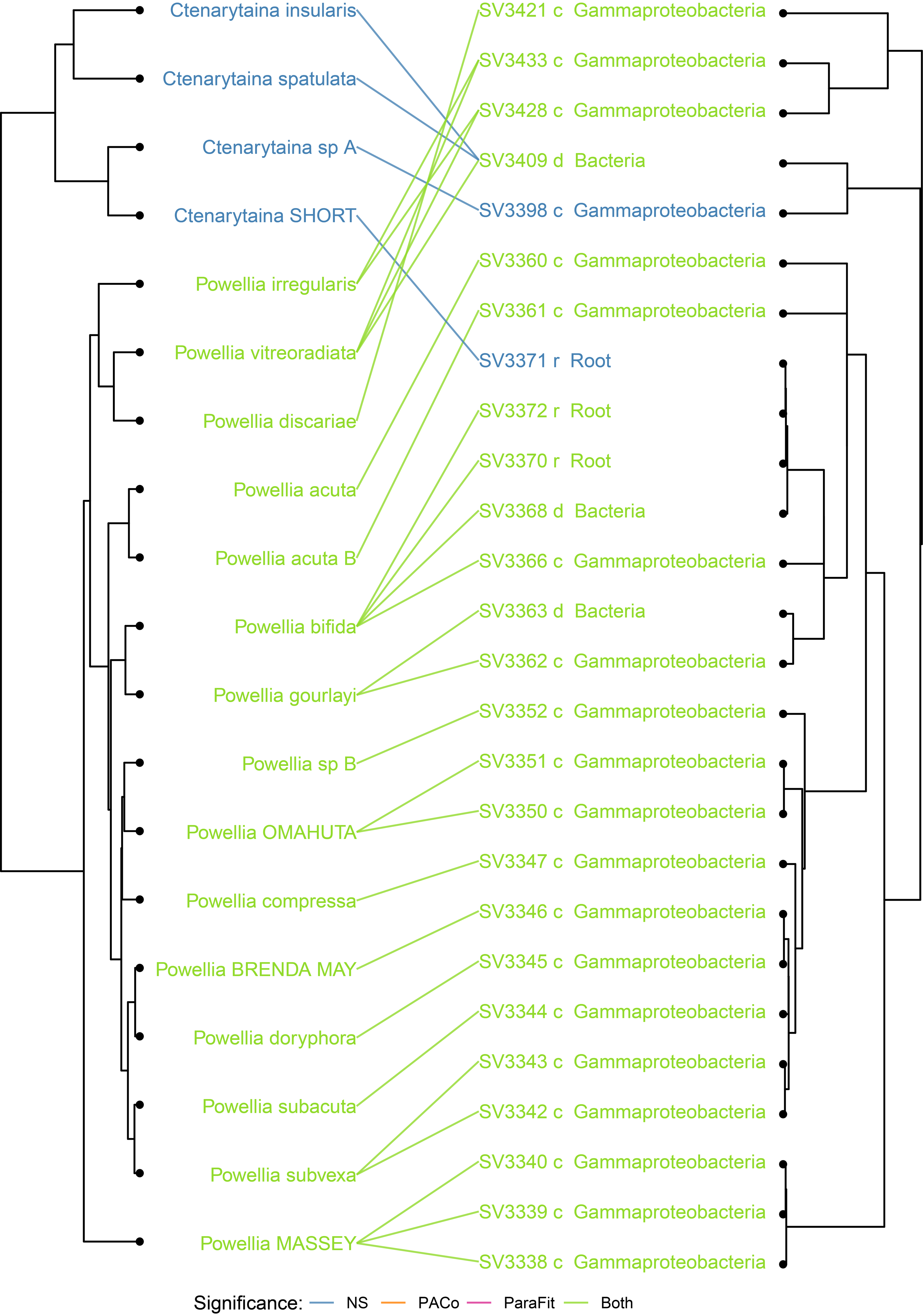

Supplement: S5 Fig — Links and taxa coloured by whether significant co-phylogenetic signal was found with PACo or ParaFit. (TIF) [file pone.0285587.s005.TIF]

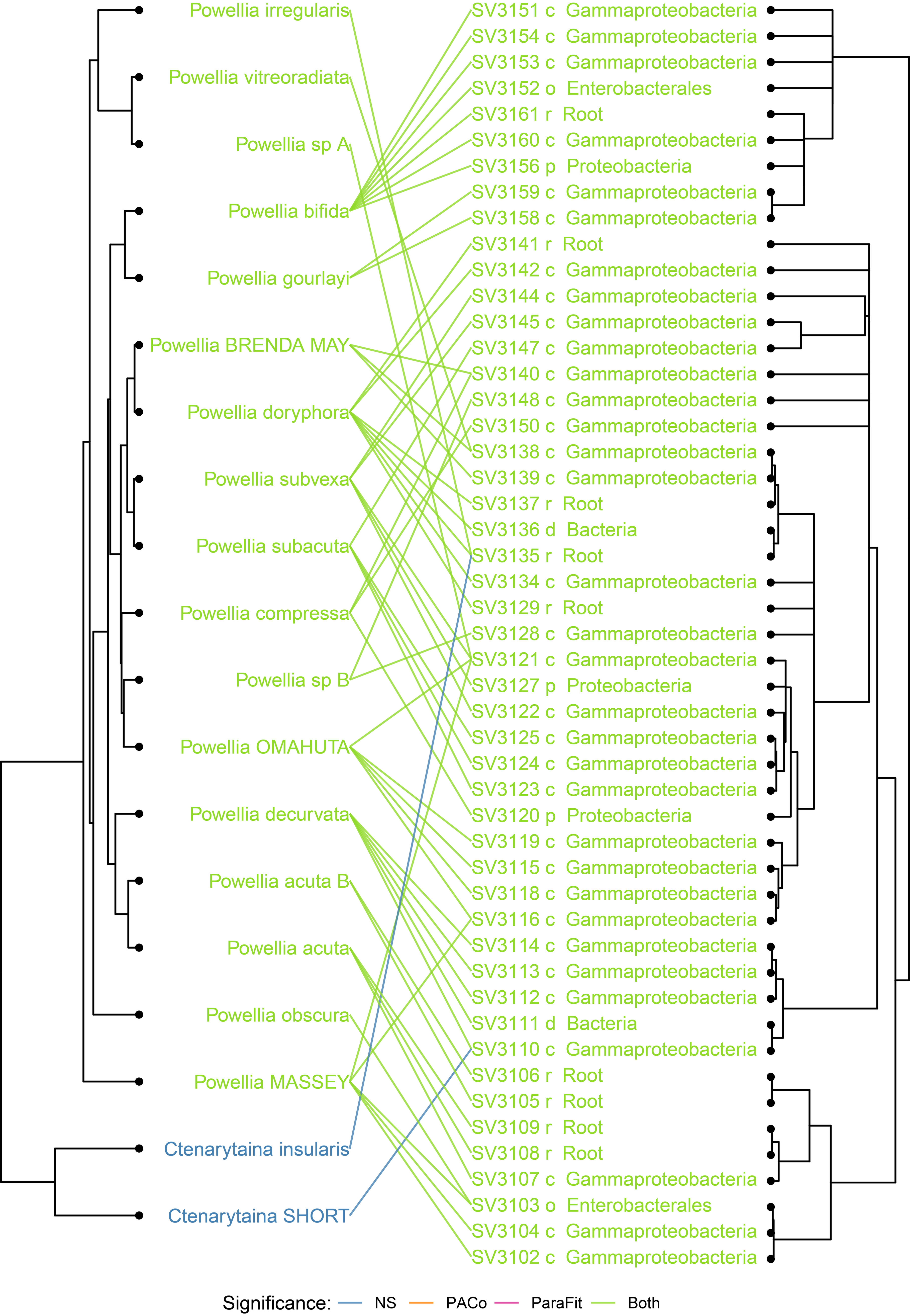

Supplement: S6 Fig — Links and taxa coloured by whether significant co-phylogenetic signal was found with PACo or ParaFit. (TIF) [file pone.0285587.s006.TIF]

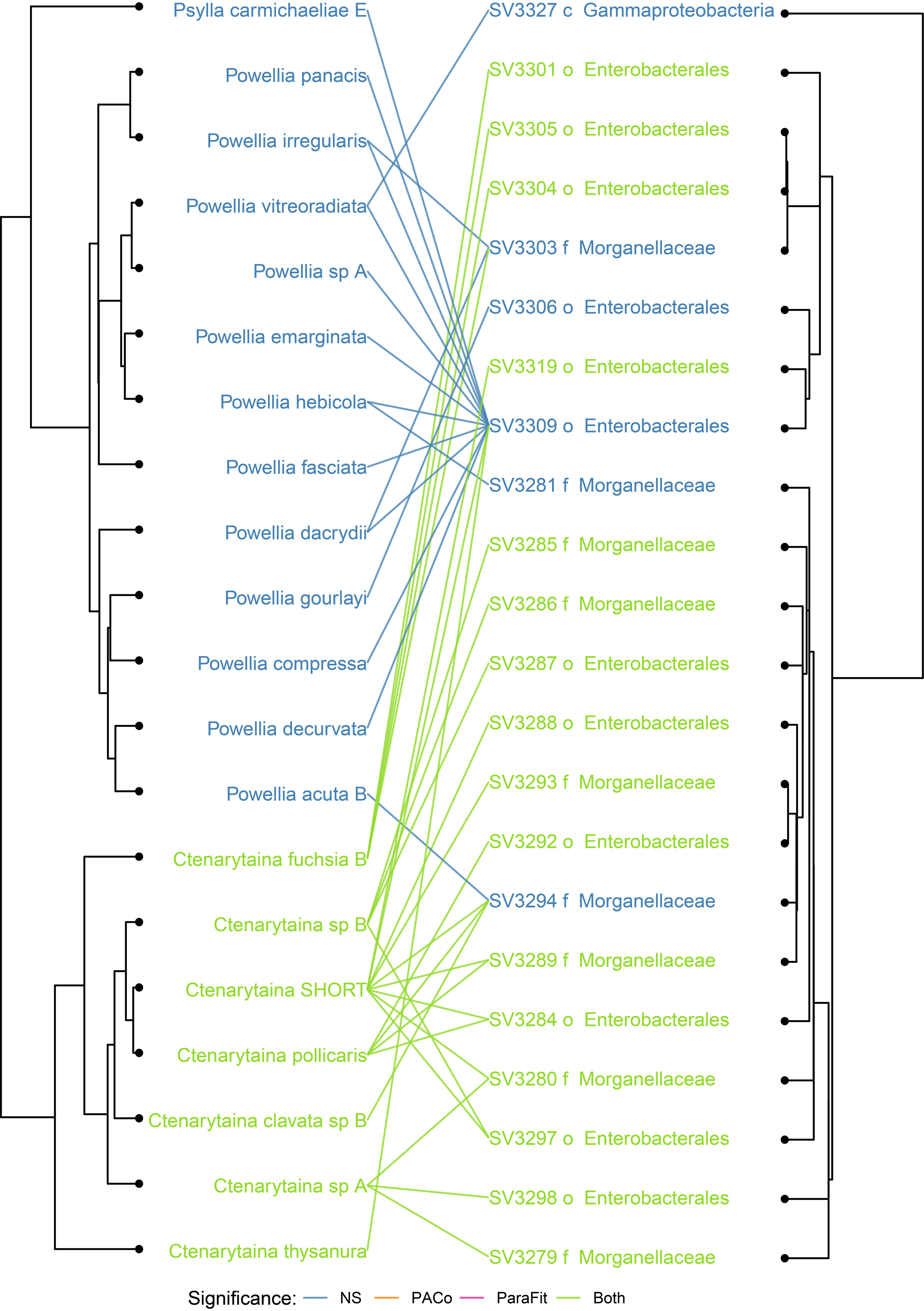

Supplement: S7 Fig — Links and taxa coloured by whether significant co-phylogenetic signal was found with PACo or ParaFit. (TIF) [file pone.0285587.s007.TIF]

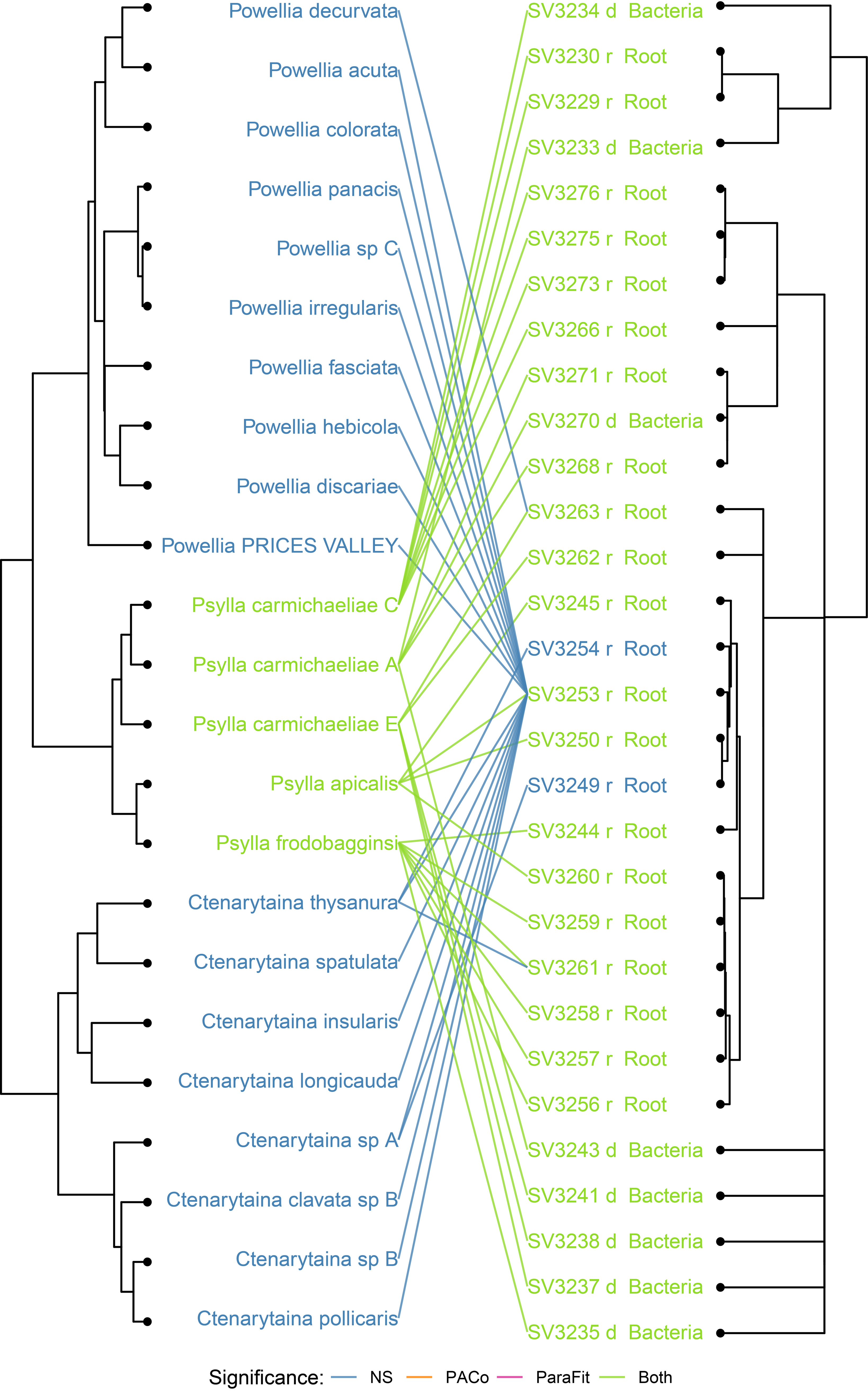

Supplement: S8 Fig — Links and taxa coloured by whether significant co-phylogenetic signal was found with PACo or ParaFit. (TIF) [file pone.0285587.s008.TIF]

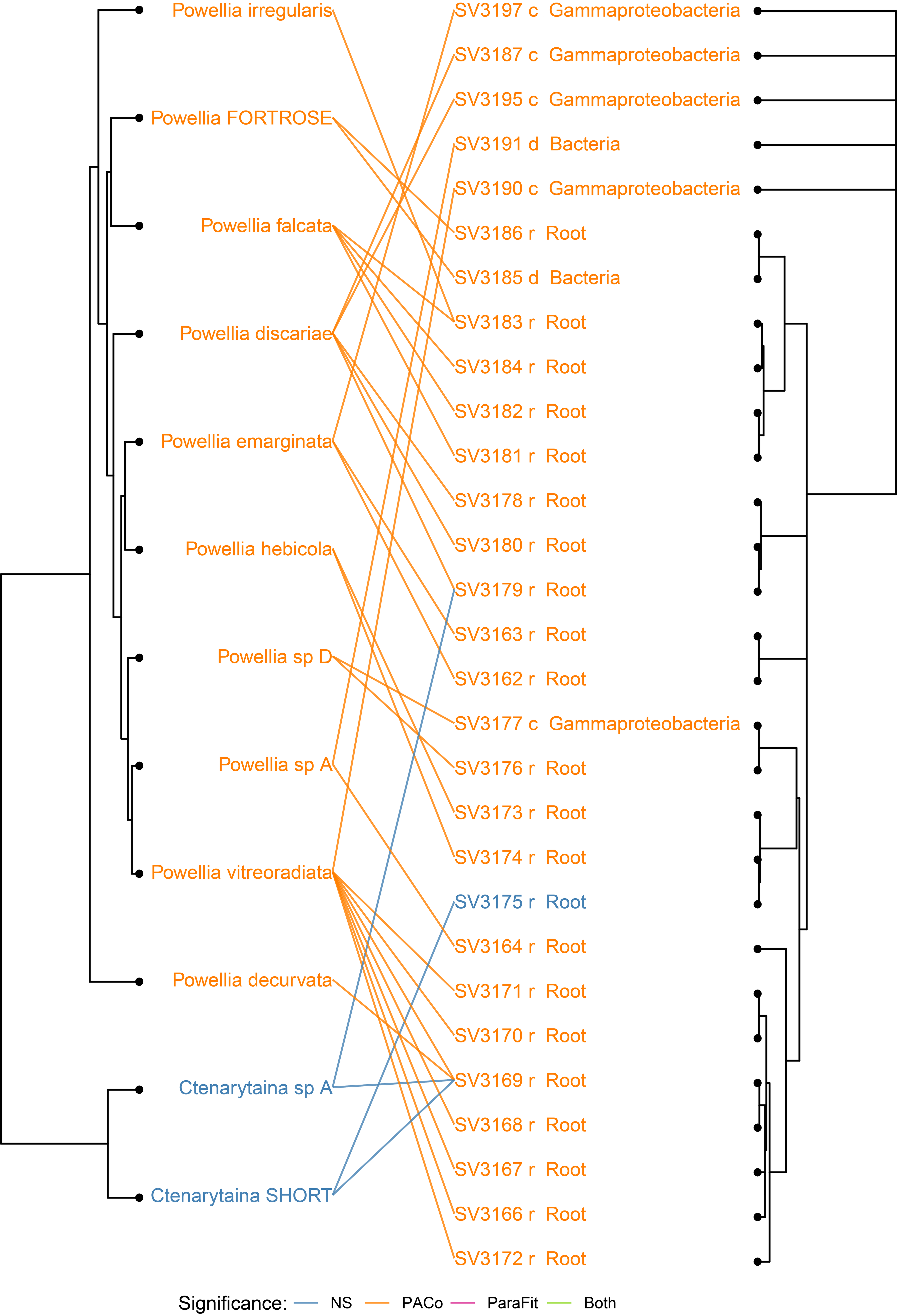

Supplement: S9 Fig — Links and taxa coloured by whether significant co-phylogenetic signal was found with PACo or ParaFit. (TIF) [file pone.0285587.s009.TIF]

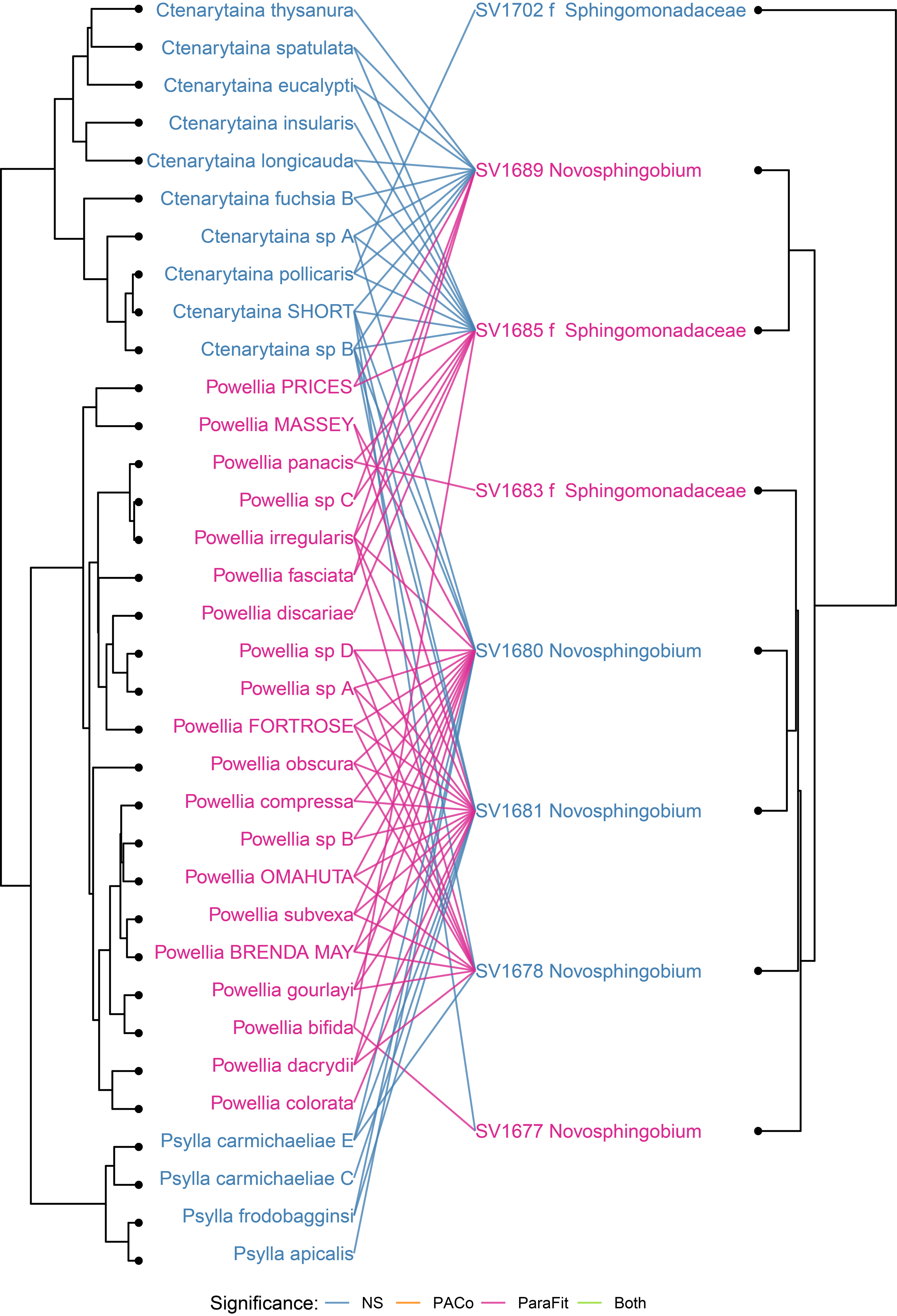

Supplement: S10 Fig — Links and taxa coloured by whether significant co-phylogenetic signal was found with PACo or ParaFit. (TIF) [file pone.0285587.s010.TIF]

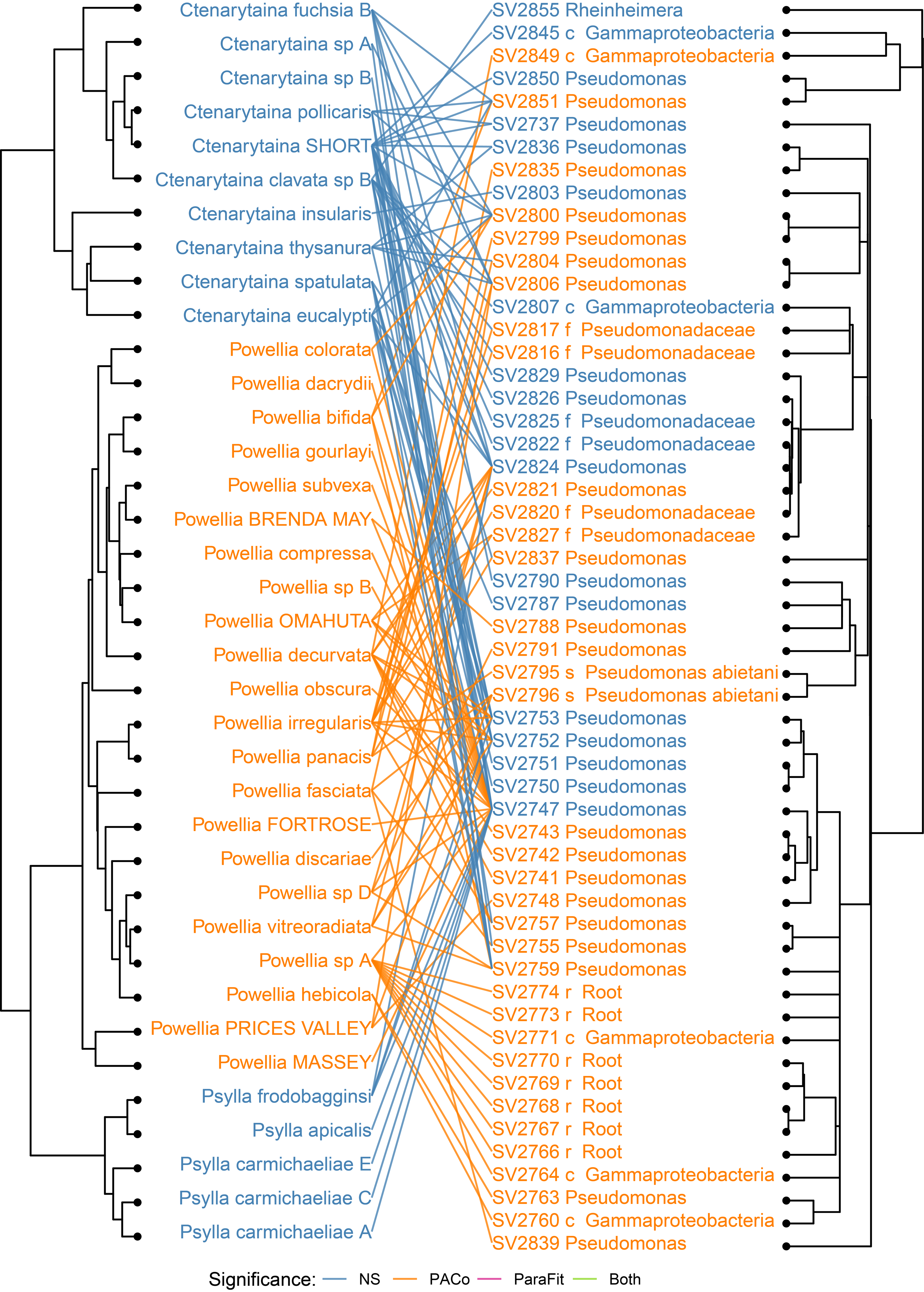

Supplement: S11 Fig — Links and taxa coloured by whether significant co-phylogenetic signal was found with PACo or ParaFit. (TIF) [file pone.0285587.s011.TIF]
