# Supplementary material for: Insect phylogeny structures the bacterial communities in the microbiome of psyllids (Hemiptera: Psylloidea) in Aotearoa New Zealand
Source: PLoS One. 2023 May 15;18(5):e0285587. doi: 10.1371/journal.pone.0285587 (PMC10184942; doi:10.1371/journal.pone.0285587)
Supplement: S1 Table — The table lists the species analysed and the family they belong to. Information on the endemicity to New Zealand of the species is reported for invasive (I), native (N), endemic (E) and non-present (NP) species. Collection location for the samples is provided together with the number of samples and populations analysed in this study. The number of DNA sequences used is reported together with accession numbers for the COI, EF-1α and 18S genes. Accession numbers in bold are for the sequences generated in this study. (DOCX) [file pone.0285587.s012.docx]

**Table SM1:** **Psyllid samples used in this study for insect phylogenetic analysis.** The table lists the species analysed and the family they belong to. Information on the endemicity to New Zealand of the species is reported for invasive (I), native (N), endemic (E) and non-present (NP) species. Collection location for the samples is provided together with the number of samples and populations analysed in this study. The number of DNA sequences used is reported together with accession numbers for the COI, EF-1α and 18S genes. Accession numbers in bold are for the sequences generated in this study.

| **Family** | **Species** | **Endemicity** | **Location** | **Samples** | **Populations** | **COI** | **18S** | **EF-1α** |
| --- | --- | --- | --- | --- | --- | --- | --- | --- |
| Psyllidae | *Acizzia acaciae* | I | NZ | 8 | 7 | 8 [MG132227.1 - MG132234.1] | **2 [MG195289.1 - MG195290.1]** |  |
|  | *Acizzia acaciaebaileyanae* | I | NZ/AUS | 6 | 2 | 6 [MG132271.1 - MG132276.1] | **1 [MG195305.1]** |  |
|  | *Acizzia albizziae* | I | NZ | 14 | 12 | 14 [MG132235.1 - MG132247.1; MF197452] | **3 [MG195291.1 - MG195293.1]** | 1 [KY983277.1] |
|  | *Acizzia conspicua* | I | NZ | 2 | 1 | 2 [MG132248.1 - MG132249.1] | **2 [MG195294.1 - MG195295.1]** |  |
|  | *Acizzia dodonaeae* | I | NZ | 10 | 8 | 10 [MG132250.1 - MG132259.1] | **2 [MG195296.1 - MG195297.1]** |  |
|  | *Acizzia errabunda* | I | NZ/AUS | 6 | 3 | 6 [MG132221.1 - MG132226.1] | **1 [MG195288.1]** |  |
|  | *Acizzia exquisita* | I | NZ | 2 | 1 | 2 [MG132260.1 - MG132261.1] | **2 [MG195298.1 - MG195299.1]** |  |
|  | *Acizzia hakeae* | I | NZ | 4 | 4 | 4 [MG132262.1 - MG132265.1] | **2 [MG195300.1 - MG195301.1]** |  |
|  | *Acizzia jucunda* | I | NZ | 5 | 3 | 5 [MG132266.1 - MG132270.1] | **2 [MG195302.1 - MG195303.1]** |  |
|  | *Acizzia solanicola* | I | NZ | 1 | 1 |  | **1 [MG195304.1]** |  |
|  | *Acizzia uncatoides* | I | NZ/AUS | 18 | 16 | 18 [MG132277.1 - MG132294.1] | **3 [MG195306.1 - MG195308.1]** |  |
|  | *Acizzia* 'Waitakere’ | E | NZ | 2 | 2 | 2 [MG132295.1 - MG132296.1] | **1 [MG195309.1]** |  |
|  | *Arytainilla spartiophila* | I | NZ | 4 | 4 | 4 [MG132304.1 - MG132307.1] | **2 [MG195316.1 - MG195317.1]** |  |
|  | *Diaphorina citri* | NP | USA | 2 | 1 | 2 [MG132457.1 - MG132458.1] | 3 [MG195376.1 - MG195378.1] |  |
|  | *Heteropsylla texana* | NP | USA | / | / | 1 [MG988760.1] | 1 [MG988595.1] |  |
|  | *Psylla alni* | NP | EU | / | / | 3 [MG988817.1, KU517189.1, MG989235.1] | 1 [MG988605.1] |  |
|  | *Psylla apicalis* | E | NZ | 6 | 5 | 6 [MG132465.1 - MG132470.1] | **1 [MG195385.1]** |  |
|  | *Psylla foersteri* | I | NZ | 2 | 2 | 2 [MG132313.1; MF197458] | 2 [MG195323.1 - MG195324.1] | 1 [KY983275.1] |
|  | *Psylla frodobagginsi* | E | NZ | 11 | 11 | 11 [MG132471.1 - MG132481.1] | **1 [MG195386.1]** |  |
|  | *Psylla buxi* | NP | EU | / | / | 2 [KU517188.1, MG988818.1] | 1 [MG988606.1] |  |
|  | *Psylla carmichaeliae* A | E | NZ | 7 | 4 | 7 [MG132482.1 - MG132488.1] | **3 [MG195387.1 - MG195389.1]** |  |
|  | *Psylla carmichaeliae* B | E | NZ | 3 | 1 | 3 [MG132489.1 - MG132491.1] | **1 [MG195390.1]** |  |
|  | *Psylla carmichaeliae* C | E | NZ | 3 | 3 | 3 [MG132492.1 - MG132494.1] | **1 [MG195391.1]** |  |
|  | *Psylla carmichaeliae* D | E | NZ | 1 | 1 | 1 [MG132495.1] | **1 [MG195392.1]** |  |
|  | *Psylla carmichaeliae* E | E | NZ | 5 | 3 | 5 [MG132496.1 -MG132500.1] | **2 [MG195393.1 - MG195394.1]** |  |
| Triozidae | *Acanthocasuarina acutivalvis* | NP | AU | 1 | 1 | **1 [MK284246.1]** | **1 [MK284245.1]** |  |
|  | *Bactericera cockerelli* | I | NZ | 3 | 2 | 3 [MG132308.1 - MG132309.1; MF197456] | **1 [MG195320.1]** |  |
|  | *Bactericera dorsalis* | NP | USA | 3 | 1 | 3 [MG132310.1 - MG132312.1] | **2 [MG195321.1 - MG195322.1]** |  |
|  | *Casuarinicola australis* | I | NZ/AUS | 3 | 2 | 3 [MG132321.1; MG132322.1; MF197461] | **1 [MG195331.1]** | 1 [KY983276.1] |
|  | *Powellia acuta* A | E | NZ | 4 | 3 | 4 [MG132507.1 - MG132510.1] | **1 [MG195399.1]** |  |
|  | *Powellia acuta* B | E | NZ | 1 | 1 | 1 [MG132598.1] | **1 [MG195449.1]** |  |
|  | *Powellia bifida* | E | NZ | 13 | 11 | 13 [MG132514.1 - MG132526.1] | **3 [MG195402.1 - MG195404.1]** |  |
|  | *Powellia* 'Brenda May' | E | NZ | 2 | 2 | 2 [MG132527.1 - MG132528.1] | **1 [MG195405.1]** |  |
|  | *Powellia colorata* | E | NZ | 4 | 3 | 4 [MG132529.1 - MG132532.1] | **2 [MG195406.1 - MG195407.1]** |  |
|  | *Powellia compressa* | E | NZ | 4 | 3 | 4 [MG132533.1 - MG132536.1] | **3 [MG195408.1 - MG195410.1]** |  |
|  | *Powellia dacryidii* | E | NZ | 2 | 1 | 2 [MG132539.1 - MG132540.1] | **2 [MG195413.1 - MG195414.1]** |  |
|  | *Powellia decurvata* | E | NZ | 3 | 3 | 3 [MG132541.1 - MG132543.1] | **2 [MG195415.1 - MG195416.1]** |  |
|  | *Powellia discariae* | E | NZ | 4 | 3 | 4 [MG132544.1 - MG132547.1] | **2 [MG195417.1 - MG195418.1]** |  |
|  | *Powellia doryphora* | E | NZ | 4 | 4 | 4 [MG132548.1 - MG132551.1] | **2 [MG195419.1 - MG195420.1]** |  |
|  | *Powellia emarginata* | E | NZ | 1 | 1 | 1 [MG132552.1] | **1 [MG195421.1]** |  |
|  | *Powellia falcata* A | E | NZ | 4 | 4 | 4 [MG132553.1 - MG132556.1] | **2 [MG195422.1 - MG195423.1]** |  |
|  | *Powellia falcata* B | E | NZ | 1 | 1 | 1 [MG132604.1] | **1 [MG195453.1]** |  |
|  | *Powellia fasciata* | E | NZ | 2 | 1 | 2 [MG132557.1 - MG132558.1] | **2 [MG195424.1 - MG195425.1]** |  |
|  | *Powellia* 'Fortrose' | E | NZ | 2 | 1 | 2 [MG132559.1 - MG132560.1] | **2 [MG195426.1 - MG195427.1]** |  |
|  | *Powellia gourlayi* | E | NZ | 2 | 1 | 2 [MG132561.1 - MG132562.1] | **2 [MG195428.1 - MG195429.1]** |  |
|  | *Powellia hebicola* | E | NZ | 2 | 1 | 2 [MG132563.1 - MG132564.1] | **1 [MG195430.1]** |  |
|  | *Powellia irregularis* | E | NZ | 11 | 10 | 11 [MG132565.1 - MG132575.1] | **2 [MG195431.1 - MG195432.1]** |  |
|  | *Powellia* 'Massey' | E | NZ | 4 | 2 | 4 [MG132576.1 - MG132579.1] | **3 [MG195433.1 - MG195435.1]** |  |
|  | *Powellia obscura* | E | NZ | 3 | 2 | 3 [MG132580.1 - MG132582.1] | **3 [MG195436.1 - MG195438.1]** |  |
|  | *Powellia* 'Omahuta' | E | NZ | 3 | 3 | 3 [MG132583.1 - MG132585.1] | **2 [MG195439.1 - MG195440.1]** |  |
|  | *Powellia panacis* | E | NZ | 4 | 3 | 4 [MG132586.1 - MG132589.1] | **1 [MG195441.1]** |  |
|  | *Powellia* 'Price’s Valley' | E | NZ | 3 | 2 | 3 [MG132590.1 - MG132592.1] | **2 [MG195442.1 - MG195443.1]** |  |
|  | *Powellia* sp. A | E | NZ | 5 | 3 | 5 [MG132593.1 - MG132597.1] | **5 [MG195444.1 - MG195448.1]** |  |
|  | *Powellia* sp. B | E | NZ | 1 | 1 | 1 [MG132599.1] | **1 [MG195450.1]** |  |
|  | *Powellia* sp. C | E | NZ | 2 | 1 | 2 [MG132600.1 - MG132601.1] | **1 [MG195451.1]** |  |
|  | *Powellia* sp. D | E | NZ | 2 | 1 | 2 [MG132602.1 - MG132603.1] | **1 [MG195452.1]** |  |
|  | *Powellia subacuta* | E | NZ | 5 | 3 | 5 [MG132605.1 - MG132609.1] | **2 [MG195454.1 - MG195455.1]** |  |
|  | *Powellia subvexa* | E | NZ | 5 | 4 | 5 [MG132610.1 - MG132614.1] | **3 [MG195456.1 - MG195458.1]** |  |
|  | *Powellia vitreoradiata* | N | NZ | 16 | 16 | 16 [MG132615.1 - MG132630.1] | **2 [MG195459.1 - MG195460.1]** |  |
|  | *Trioza adventicia* | I | NZ | 3 | 2 | 3 [MG132511.1 - MG132513.1] | **2 [MG195400.1 - MG195401.1]** |  |
|  | *Trioza apicalis* | NP | EU | / | / | 1[MG988840.1] | **2 [OM642538 - OM642539]** |  |
|  | *Trioza curta* | E | NZ | 2 | 1 | 2 [MG132537.1 - MG132538.1] | **2 [MG195411.1 - MG195412.1]** |  |
|  | *Trioza elaeagni* | NP | EU | / | / | 1[MG988842.1] | 1[MG988611.1] |  |
|  | *Trioza remota* | NP | EU | / | / | 1 [KY294163.1] | 1 [MG988613.1] |  |
|  | *Trioza tricornuta* | NP | AU | 4 | 4 | **4 [MK284247.1 - MK284250.1]** | **4 [MK284241.1 - MK284244.1]** |  |
|  | *Trioza urticae* | NP | EU | / | / | 1 [MG989240.1] | 1 [MG988614.1] |  |
|  | Triozid sp. | I | NZ/AUS | 3 | 3 | 3 [MG132323.1 - MG132325.1] | **2 [MG195332.1 - MG195333.1]** |  |
| Liviidae | *Psyllopsis fraxini* | I | NZ/AUS | 3 | 3 | 3 [MG132501.1 - MG132502.1; MF197471] | **2 [MG195395.1 - MG195396.1]** | 1 [KY983269.1] |
|  | *Psyllopsis fraxinicola* | I | NZ/AUS | 4 | 4 | 4 [MG132503.1 - MG132506.1] | **2 [MG195397.1 - MG195398.1]** |  |
| Calophyidae | *Calophya schini* | I | NZ | 4 | 3 | 4 [MG132316.1 - MG132319.1] | **2 [MG195327.1 - MG195328.1]** |  |
| Aphalaridae | *Anoeconeossa communis* | I | NZ | 3 | 1 | 3 [MG132297.1 - MG132298.1; MF197453] | **1 [MG195310.1]** | 1 [KY983266.1] |
|  | *Anomalopsylla insignita* | E | NZ | 4 | 3 | 4 [MG132299.1 - MG132301.1; MF197454] | **5 [MG195311.1 - MG195315.1]** | 1 [KY983265.1] |
|  | *Anomalopsylla* 'Pollen Island' | E | NZ | 3 | 1 | 3 [MG132302.1 - MG132303.1; MF197455] |  | 1 [KY983261.1] |
|  | *Atmetocranium myersi* | E | NZ | 2 | 1 |  | **2 [MG195318.1 - MG195319.1]** | **1 [MH556913]** |
|  | *Blastopsylla occidentalis* | I | NZ/AUS | 6 | 6 | 6 [MG132314.1 - MG132315.1; MF197459; **MK284251.1 - MK284253.1**] | **2 [MG195325.1 - MG195326.1]** | 1 [KY983268.1] |
|  | *Cardiaspina fiscella* | I | NZ/AUS | 3 | 3 | 3 [MG132320.1; MF197460; **MK284254.1**] | **2 [MG195329.1 - MG195330.1]** | 1 [KY983257.1] |
|  | *Creiis lituratus* | I | NZ | 1 | 1 | 1 [MF197462] | **1 [MG195334.1]** | 1 [KY983256.1] |
|  | *Cryptoneossa triangula* | I | NZ/AUS | 10 | 8 | 10 [MG132326.1 - MG132328.1; MF197463; **MK284255.1 - MK284260.1]** | **2 [MG195335.1 - MG195336.1]** | 1 [KY983258.1] |
|  | *Ctenarytaina clavata* A | E | NZ | 4 | 4 | 4 [MG132329.1 - MG132332.1] | **2 [MG195337.1 - MG195338.1]** |  |
|  | *Ctenarytaina clavata* B | E | NZ | 1 | 1 | 1 [MG132333.1] | **1 [MG195339.1]** |  |
|  | *Ctenarytaina clavata* C | E | NZ | 2 | 1 | 2 [MG132334.1 - MG132335.1] | **2 [MG195340.1 - MG195341.1]** |  |
|  | *Ctenarytaina clavata* D | E | NZ | 3 | 3 | 3 [MG132456.1; MG132447.1; MG132448.1] | **2 [MG195368.1 - MG195369.1]** |  |
|  | *Ctenarytaina eucalypti* | I | NZ/AUS | 2 7 | 19 | 27 [MG132336.1 - MG132361.1; MF197464] | **2 [MG195342.1 - MG195343.1]** | 1 [KY983270.1] |
|  | *Ctenarytaina fuchsia* A | E | NZ | 30 | 26 | 30 [MG132362.1 - MG132390.1; MF197465] | **5 [MG195344.1 - MG195348.1]** | 2 [KY983259.1 - KY983260.1] |
|  | *Ctenarytaina fuchsia* B | E | NZ | 7 | 1 | 7 [MG132391.1 - MG132397.1] | **1 [MG195349.1]** |  |
|  | *Ctenarytaina fuchsia* C | E | NZ | 5 | 1 | 5 [MG132398.1 - MG132402.1] | **2 [MG195350.1 - MG195351.1]** |  |
|  | *Ctenarytaina insularis* | I | NZ | 4 | 3 | 4 [MG132452.1 - MG132455.1] | **2 [MG195374.1 - MG195375.1]** |  |
|  | *Ctenarytaina longicauda* | I | NZ | 3 | 2 | 3 [MG132403.1 - MG132405.1] | **2 [MG195352.1 - MG195353.1]** |  |
|  | *Ctenarytaina pollicaris* A | E | NZ | 5 | 2 | 5 [MG132406.1 - MG132411.1] | **2 [MG195354.1 - MG195355.1]** |  |
|  | *Ctenarytaina pollicaris* B | E | NZ | 6 | 4 | 6 [MG132412.1 - MG132416.1] | **3 [MG195356.1 - MG195358.1]** |  |
|  | *Ctenarytaina* 'Short' | E | NZ | 10 | 7 | 10 [MG132417.1 - MG132425.1] | **4 [MG195359.1 - MG195362.1]** | 1 [KY983272.1] |
|  | *Ctenarytaina* sp. A | E | NZ | 1 | 1 | 1 [MG132426.1] | **1 [MG195363.1]** |  |
|  | *Ctenarytaina* sp. B | E | NZ | 2 | 2 | 2 [MG132443.1 - MG132444.1] | **1 [MG195366.1]** |  |
|  | *Ctenarytaina* sp. C | E | NZ | 2 | 2 | 2 [MG132445.1 - MG132446.1] | **1 [MG195367.1]** |  |
|  | *Ctenarytaina* sp. D | E | NZ | 1 | 1 | 1 [MG132449.1] | **1 [MG195370.1]** |  |
|  | *Ctenarytaina* sp. E | E | NZ | 2 | 1 | 1 [MG132450.1] | **2 [MG195371.1 - MG195372.1]** |  |
|  | *Ctenarytaina spatulata* | I | NZ | 17 | 17 | 17 [MG132427.1 - MG132442.1] | **2 [MG195364.1 - MG195365.1]** | 1 [KY983271.1] |
|  | *Ctenarytaina thysanura* | I | NZ | 1 | 1 | 1 [MG132451.1] | **1 [MG195373.1]** |  |
|  | *Eucalyptolyma maideni* | I | NZ | 2 | 1 | 2 [MG132459.1; MF197468] | **1 [MG195379.1]** | 1 [KY983264.1] |
|  | *Glycaspis granulata* | I | NZ/AUS | 3 | 2 | 2 [MG132460.1; MF197469] | **3 [MG195380.1 - MG195382.1]** | 1 [KY983267.1] |
|  | *Rhinocola aceris* | NP | EU | / | / | 2 [MF176157.1, MG988823.1] | 1 [MG988608.1] |  |
| Carsidaridae | *Mycopsylla fici* | I | NZ | 5 | 4 | 5 [MG132461.1 - MG132464.1; MF197470] | **2 [MG195383.1 - MG195384.1]** | 2 [KY983262.1 - KY983263.1] |
| Outgroup | *Acyrthosiphon pisum* |  |  | / | / | 1 [KR579669] | 1 [U27819.1] | 2 [XM008184147-1; XM001948705-4] |
| **Total** | **103** |  |  |  |  | **458** | **188** | **23** |
